# CRISPR/Cas9-induced modification of the conservative promoter region of *VRN-A1* alters the heading time of hexaploid bread wheat

**DOI:** 10.3389/fpls.2022.1048695

**Published:** 2022-12-05

**Authors:** Dmitry Miroshnichenko, Vadim Timerbaev, Anna Klementyeva, Alexander Pushin, Tatiana Sidorova, Dmitry Litvinov, Lubov Nazarova, Olga Shulga, Mikhail Divashuk, Gennady Karlov, Elena Salina, Sergey Dolgov

**Affiliations:** ^1^ Kurchatov Genomic Center — All-Russia Research Institute of Agricultural Biotechnology, Moscow, Russia; ^2^ Branch of Institute of Bioorganic Chemistry RAS, Pushchino, Russia; ^3^ All-Russia Research Institute of Agricultural Biotechnology, Moscow, Russia; ^4^ Institute of Cytology and Genetics, SB RAS, Novosibirsk, Russia

**Keywords:** genome editing, mutation, vernalization, *VRN-1* gene, *Triticum aestivum* L.

## Abstract

In cereals, the vernalization-related gene network plays an important role in regulating the transition from the vegetative to the reproductive phase to ensure optimal reproduction in a temperate climate. In hexaploid bread wheat (*Triticum aestivum* L.), the spring growth habit is associated with the presence of at least one dominant locus of *VERNALIZATION 1* gene (*VRN-1*), which usually differs from recessive alleles due to mutations in the regulatory sequences of the promoter or/and the first intron. *VRN-1* gene is a key regulator of floral initiation; various combinations of dominant and recessive alleles, especially *VRN-A1* homeologs, determine the differences in the timing of wheat heading/flowering. In the present study, we attempt to expand the types of *VRN-A1* alleles using CRISPR/Cas9 targeted modification of the promoter sequence. Several mono- and biallelic changes were achieved within the 125-117 bp upstream sequence of the start codon of the recessive *vrn-A1* gene in plants of semi-winter cv. ‘Chinese Spring’. New mutations stably inherited in subsequent progenies and transgene-free homozygous plants carrying novel *VRN-A1* variants were generated. Minor changes in the promoter sequence, such as 1–4 nucleotide insertions/deletions, had no effect on the heading time of plants, whereas the CRISPR/Cas9-mediated 8 bp deletion between −125 and −117 bp of the *vrn-A1* promoter shortened the time of head emergence by up to 2-3 days. Such a growth habit was consistently observed in homozygous mutant plants under nonvernalized cultivation using different long day regimes (16, 18, or 22 h), whereas the cold treatment (from two weeks and more) completely leveled the effect of the 8 bp deletion. Importantly, comparison with wild-type plants showed that the implemented alteration has no negative effects on main yield characteristics. Our results demonstrate the potential to manipulate the heading time of wheat through targeted editing of the *VRN-A1* gene promoter sequence on an otherwise unchanged genetic background.

## Introduction

Various external and internal factors can affect the transition to the reproductive phase, but the photoperiod and temperature are recognized as the most crucial (Kiseleva and Salina, 2018; Shi et al., 2019). In common wheat (*Triticum aestivum* L.), ancestral ‘winter’ forms start flowering only after prolonged exposure to low temperatures, known as the vernalization requirement. The varieties with a ‘spring’ growth habit are less sensitive to vernalization and develop spikes without prior exposure to cold. The variations in vernalization requirements are mainly regulated by epistatic interactions between a range of *VERNALIZATION* genes, such as *VRN1*, *VRN2*, *VRN3*, and *VRN4* (Xu and Chong, 2018; Sharma et al., 2020).

A central role in regulating the response to cold belongs to the *VRN1* gene, which encodes the MADS box transcription factor (Yan et al., 2003) homologous to *APETALA1*, *CAULIFLOWER*, and *FRUITFULL* (Xu and Chong, 2018; Shi et al., 2019). Without vernalization, *VRN1* is not active at the early stages of the vegetative growth phase of whiter varieties, but during exposure to low temperatures, its expression is progressively increased under short winter days. The cold-induced upregulation of *VRN1* downregulates the activity of the *VRN2* gene, known as the floral repressor (Yan et al., 2004). The products of the *VRN2* gene, zinc finger CCT domain proteins, operate as negative regulators of the activity of the *VRN3* gene, a flowering integrator (Yan et al., 2006). Under short winter days, *VRN2* is highly expressed, and transcription factors encoded by *VRN2* can competitively interact with members of the family of CCT-domain proteins, which bind with the promoter of the *VRN3* gene (Li et al., 2011) encoding polyethanolamine-binding protein, which highly homologous to *Arabidopsis FLOWERING LOCUS T* (Xu and Chong, 2018). Before vernalization, *VRN3* demonstrates a low level of expression, but the increase in *VRN1* activity during vernalization suppresses *VRN2*. Lowering the levels of *VRN2* abolishes the repression of *VRN3* genes. The activation of *VRN3* in leaves resulting from the increased day length in spring additionally stimulates *VRN1* expression, thus forming a positive-feedback regulatory loop (Xu and Chong, 2018; Shi et al., 2019). The expression of *VRN1*, after reaching a certain threshold of transcripts, ensures the successful transition from the vegetative to the generative stage, positively affecting wheat flowering. The *VRN4* genes, which are supposed to be additional copies of the *VRN1* locus, also encode MADS box transcription factors and act similarly to the *VRN1* genes as part of the *VRN1*/*VRN2*/*VRN3* feedback loop (Kippes et al., 2015).

Common wheat, being a hexaploid cereal species, carries three homeologous copies of the *VRN1* locus designated as *VRN-A1*, *VRN-B1*, and *VRN-D1*. Winter wheat is characterized by the presence of the three recessive *vrn-A1*, *vrn-B1*, and *vrn-D1* alleles, whereas spring wheat has at least one *VRN1* gene homolog with a dominant allele (*Vrn-A1*, *Vrn-B1*, or *Vrn-D1*). The results of extensive molecular studies reveal that the dominant alleles of *VRN-1* in wheat contain insertions and deletions within the promoter or a deletion within intron 1 (Fu et al., 2005; Li et al., 2013; Konopatskaia et al., 2016; Muterko et al., 2016). For this reason, the spring varieties demonstrate constitutive expression of *VRN1*, which allows plants to bypass the vernalization requirement. The ‘spring-habit’ strength of *Vrn-1* alleles, however, is not equal and can be ranked as *Vrn-A1* > *Vrn-B1* > *Vrn-D1* (Zhang et al., 2008). Varieties with various combinations of *Vrn-1*/*vrn-1* alleles differ significantly regarding the vernalization requirement. The semi-winter varieties carrying recessive *vrn-A1* and dominant *Vrn-B1* or *Vrn-D1* alleles are able to develop spikes without vernalization (intermediate growth habit), but in the case of exposure to cold, the transition from the vegetative to the reproductive stage is shorter due to the activation of *VRN-A1*.

Recent progress regarding knowledge of the structure and function of wheat *VRN-1* genes indicates that *VRN-A1* dominant alleles predominantly contain mutations located in the promoter sequence, whereas the dominant alleles of *VRN-B1* and *VRN-D1* mostly carry deletions within the first intron (Konopatskaia et al., 2016; Muterko et al., 2016; Shi et al., 2019; Strejčková et al., 2021). The promoters of wheat *VRN-A1* genes contain various conservative regulatory cis elements, such as CArG, VRN, and G boxes, which significantly influence the functional activity of *VRN-1* (Kiseleva and Salina, 2018). To date, the region of 16 bp (“TTAAAAACCCCTCCCC”), the so-called ‘VRN box’, is regarded to have the greatest impact on the ‘winter-spring’ growth habit of wheat (Shi et al., 2019). A number of nucleotide modifications of the VRN box have been described in polyploid wheat species. In common wheat, the dominant alleles of *VRN-A1* include *Vrn-A1a* (*Vrn-A1a.1*), *Vrn-A1a.2*, *Vrn-A1b* (*VrnA1b.1*), *Vrn-A1b.2*, *Vrn-A1b.5*, *Vrn-A1b.6*, and *Vrn-A1c* (Konopatskaia et al., 2016; Shi et al., 2019). The most common and strongest dominant allele, *Vrn-A1a.1*, differs from the recessive allele *vrn-A1* on the basis of having inserts of repeated sequences of a miniature inverted-repeat transposable element (MITE, 222 bp) in the VRN box region (Yan et al., 2004; Fu et al., 2005). A variant of *Vrn-A1a* known as *Vrn-A1a.2* additionally includes a 16 bp deletion and four single nucleotide deletions within the MITE (Muterko et al., 2016). Allelic variants of *Vrn-A1b* are characterized by the 20 bp deletion at −157 bp, whereas the difference between *Vrn-A1b.1*, *Vrn-A1b.2*, *Vrn-A1b.5*, *Vrn-A1b.6* alleles is determined by specific mutations (deletion and substitutions) within A-tract and C-rich regions of the VRN box (Muterko et al., 2016). The other dominant allele reported in the common wheat, *Vrn-A1c*, has no modifications in the promoter sequence but possesses a deletion in the first intron (Fu et al., 2005).

In less widespread wheat species, changes in the sequence of *VRN-A1* promoter associated with spring habit have also been detected (Konopatskaia et al., 2016; Muterko and Salina 2018; Shi et al., 2019), including deletions of different lengths in the VRN box (*Vrn-A1d*, *Vrn-A1e*), CarG box (*Vrn-A1e*), and G box (*Vrn-A1f*). The dominant alleles of spring tetraploid *T. turgidum* wheat species are known to carry large deletions in the promoter sequence partially covering the CarG box, such as a 32 bp deletion (*Vrn-A1d*) and a 54 bp deletion (*Vrn-A1e*). It is currently assumed that the CarG motif itself, however, does not participate in the vernalization response in wheat, as vernalization sensitivity is still retained following its complete deletion (Pidal et al., 2009). In contrast to most known dominant alleles of *T. aestivum* associated with mutations in the VRN box, some wheat species carry mutations located 50–70 bp of the VRN box. Large deletion of 50 bases (in the area between −62 and −112 bp) and the minor deletion of 8 bases (in the G box) was discovered in the *Vrn-A1f* allele of spring accessions of *T.monococcum* (Dubcovsky et al., 2006) and *T. timopheevii* (Golovnina et al., 2010). The large 42 bp fragment insertion located at −122 has been described in the dominant *Vrn-A1k* allele of spring *T. dicoccum* (Muterko and Salina, 2017). The dominant allele *Vrn-A1j*, which contains the deletion of 54 bases between −140 and −87 relative to the transcription initiation site, was recently identified in *T. compactum* (Muterko and Salina, 2018). The mutations located in the promoter regions of the *Vrn-A1f*, *Vrn-A1k*, and *Vrn-A1j* alleles do not overlap with most known VRN box-related mutations, and this makes them very interesting subjects for both practical and fundamental studies.

Transferring wild mutated *VRN-A1* alleles into the common wheat genome without changing the genetic background is not an easy task. Traditional mutagenesis and crossbreeding strategies are quite laborious and time-consuming. The methods of genetic engineering are also not fully applicable. Overexpression or RNA interference strategies cannot be used because they involve the coding sequence genes and not the promoter. Synchronous introduction of the mutated promoter and gene sequence also does not allow precisely determining their impact, since the genome of the resulting transgenic plants still carries the original *VRN-A1* alleles. The fast development of the CRISPR/Cas9 genome editing technology opens the possibility to modify specific nucleotide sequences and produce plants carrying new mutations without making any changes in the rest of the genome (Li et al., 2021). This technology is ideally suited for analyzing the functional activity of specific promoters, since it allows modifying the promoter sequence while keeping the coding sequence of the gene untouched.

The objective of our study was to generate common wheat plants carrying new mutations in the promoter regions of the recessive *vrn-A1* gene with the help of CRISPR/Cas9 tools. Various sgRNAs have been designed to target two loci, one being the sequence of the VRN box, the other the 50 bp region located between −62 and −112 bp of the promoter. Using this strategy, several wheat lines carrying the new mutations were produced, and their representative transgene-free homozygous progenies were analyzed for characterization of the heading date.

## Materials and methods

### Plant materials

The experiments were performed using a near-isogenic line of the semi-winter common wheat variety ‘Chinese Spring’ that is homozygous for recessive *vrn-A1* and *vrn-B1* alleles and homozygous for the dominant *Vrn-D1* allele. Donor wheat plants were grown in a temperature-controlled greenhouse (18–25°C) under a 16 h photoperiod with additional lighting (up to 150 lmol/m^−2^ s^−1^).

### Target locus selection and sgRNA design

The fragment of the promoter region of the *vrn-A1* gene was amplified from the genomic DNA of ‘Chinese Spring’ using the primers listed in **Supplementary Table S1**. The PCR products were digested using *Eco*RI and *Kpn*I (Thermo Fisher Scientific, USA), cloned at the corresponding sites in the pUC18 vector, and sequenced. The sequence of the *vrn-A1* promoter region is shown in **Supplementary Figure S1**. The potential targets were selected for the design of candidate sgRNAs in the two regions of the *vrn-A1* promoter. They include the VRN box region (yellow letters) and 59 bp sequence located between −62 and −121 bp (green letters) (**Supplementary Figure S1**). Potential targets for sgRNAs were selected using available algorithms and programs (Doench et al., 2016).

### *In vitro* cleavage assay

The cleavage templates were made by adding nucleotides sequences of TTC TAA TAC GAC TCA CTA TAG and GTT TTA GAG CTA GAA ATA GC to the 5’-end the 3’-end of each predicted sgRNA sequence (without PAM), mixed with primer listed in **Supplementary Table S1**, and amplified. The RNA of 14 sgRNAs was then synthesized from amplified template DNA using HiScribe T7 Quick High Yield RNA Synthesis Kit (New England Biolabs) according to the manufacturer’s instructions. Synthesized sgRNA (30 ng) and 30 ng of Cas9 nuclease (NEB) were mixed in a volume of 30 µL and incubated for 10 min at room temperature to allow the assembly of RNP complexes. The mix was then incubated for 2 h at 37 °C in NEB reaction buffer with 3 nM of the plasmid (linearized with *Ssp*I) carrying the target sequence. The reaction was stopped by adding 1 µL of proteinase K (30 mg/mL), and the digestion products were separated on a 1.2% agarose gel.

### Construction of CRISPR/Cas9 gene editing vectors

**Figure 1:**
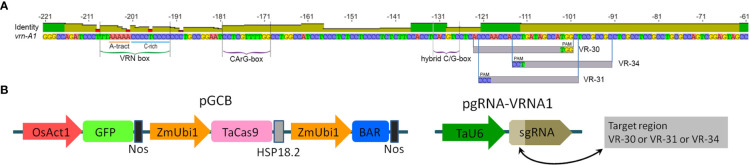
**(A)** Structure of the recessive *vrn-A1* promoter sequence of ‘Chinese Spring’ and target locations used for sgRNA vector design. **(B)** Schematic structure of linearized Cas9 and sgRNA cassettes used for CRISPR/Cas9 editing.

The Cas9 expression vector pGCB (**Figure 1B**) is based on the psGFP-BAR plasmid harboring the green fluorescent protein gene (GFP) driven by the rice *Act1* promoter and the BAR gene (phosphinothricin acetyltransferase) under the maize *Ubi1* promoter (Richards et al., 2001). A codon-optimized sequence of Cas9 driven by the maize *Ubi1* promoter was cloned from the pTaCas9 plasmid (the plasmid was a gift from Daniel Voytas) (Čermák et al., 2017; Addgene plasmid # 91169) and assembled into the psGFP-BAR between the *GFP* and BAR genes using standard cloning methods.

To construct the target-specific sgRNA vectors, the 20 bp oligos (vr-30, vr-31, and vr-34) were synthesized and cloned into the *Bpi*I site of the plasmid pU6-gRNA (Addgene plasmid #53062). The successful construction of the three vectors (pgRNA-VRNA1#30, pgRNA-VRNA1#31, and pgRNA-VRNA1#34) harboring the corresponding candidate sgRNA sequence under the *U6* promoter was confirmed by Sanger sequencing, and they were separately used for co-transformation with pGCB (1:1, v/v).

### Wheat transformation

The biolistic-mediated transformation was performed with the help of a particle inflow gun using 7–15-day-old embryogenic calli induced from isolated immature embryos as earlier described (Miroshnichenko et al., 2020). To avoid the formation of escapes, a dual selection strategy was used that is based on the continuous visual selection of transgenic tissues/plants displaying *GFP* expression on a phosphinotricin-enriched medium.

### Genotyping of transgenic plants

Genomic DNA was isolated from the leaf tissue of T0 transgenic plants using the cetyltrimethylammonium bromide (CTAB) method. The integration of Cas9 and sgRNA cassettes was examined by PCR using specific primers listed in **Supplementary Table S1**. For assessing mutagenesis, the genomic regions surrounding the target sites of *vrn-A1*, *vrn-B1*, and *vrn-D1* promoters were PCR-amplified using the corresponding primers. The PCR amplicons were sequenced with an Illumina platform through a commercial service provided by Evrogen JSC (Russia). To identify cis-regulatory elements that could be potentially disrupted by induced mutations, the relevant part of the *vrn-A1* gene promoter was analyzed using the PLACE (Higo et al., 1999; http://www.dna.affrc.go.jp/PLACE/) and PlantCARE databases (Lescot et al., 2002; http://bioinformatics.psb.ugent.be/webtools/plantcare/html/).

### Heading time evaluation

To determine the days to heading, the experimental plants (T3/M3 homozygous populations) were grown in a photoperiod-controlled glasshouse without vernalization treatment at 16-18°C/19-22°C (night/day) under a long photoperiod (16 h light). Plants were grown in 15 cm pots, with three plants per pot, using a randomized design and 6 to 24 replicates (pots) depending on seed availability. Heading time for each plant was recorded when the primary spike was fully emerged from the flag leaf sheath. Flowering time was recorded when the first anthers were visible on the primary spike.

Three GE lines carrying various deletions on the promoter were tested in climate chambers conditioned with long days during the whole life cycle. Two photoperiod regimes were used: 18 h for light at 22 ± 0.5°C, and 22 h for light at 17 ± 0.5°C). Five replicates (1.5 L pots) with five plants per pot were grown using a randomized design in a ‘Fitotron’ climate chamber (Weiss Technik, Germany).

For the vernalization experiments, M4 plants were grown in a greenhouse at 18-20°C/22-24°C (night/day) until the appearance of the third leaf (15 days) and were then transferred into a growth chamber for 15, 30, and 45 days and vernalized at 4-6 °C under a 12 h day length regime. A proportion of plants was left in the greenhouse as the unvernalized control. After this period, both vernalized and unvernalized plants were grown in a greenhouse at 18-20°C/22-24°C (night/day) under a long photoperiod (16 h light).

### Automated digital phenotyping

Digital phenotyping was carried out using the Trait finder system equipped with two PlantEye 3D scanners (Phenospex, Netherlands) by scanning the surface area of experimental plants with a resolution of less than 1 mm^3^ and measuring the volume of digital biomass and the reflection of light in several spectra bands including blue (460-485 nm), green (530-540 nm), red (620-645 nm), and near-infrared (720-750 nm) spectral regions. To detect potential variations in the spectral properties of wheat plants, the normalized difference vegetation index (NDVI) and plant senescence reflectance index (PSRI) were measured from the seedling to tillering stage of plants based on four repeated measurements of individual pots at each time point.

### RNA extraction and qPCR assay

Total RNA was extracted from frozen plant tissues using TRIzol RNA Isolation Reagent (Thermo Fisher Scientific). The middle part of the fifth leaf was used for analysis. The cDNA was reverse-transcribed from 2 μg of total RNA using RevertAid Reverse Transcriptase (Thermo Scientific). qPCR was performed using SYBR Green qPCR SuperMix (Thermo Fisher Scientific) on a QuantStudio™ 5 Real-Time PCR Cycler (Thermo Fisher Scientific) following the manufacturer’s instructions. The wheat *TaWIN1* gene (Tenea et al., 2011) was selected as the internal control. The qPCR analyses were performed on three technical replicates and analyzed using QuantStudio TM Design and Analysis (Applied Biosystems, Thermo Fisher Scientific). The primers used for qPCR are listed in **Supplementary Table S1**.

### Grain quality tests

Grain protein and starch content were measured by infrared reflectance spectroscopy using InfraLUM FT-12 analyzer (Lumex, Russia).

### Statistical analysis

To determine statistical significance levels, the ANOVA procedure followed by Turkey’s multiple range test was performed for comparison of heading time and relative expression. Significant differences in the NDVI, PSRI, and grain content between lines were determined *via* t-test with p<0.05 as a threshold.

## Results

### Target selection, sgRNA design, and vector construction

Two promoter regions of the recessive *vrn-A1* gene from the semi-winter hexaploid wheat ‘Chinese Spring’ were chosen as targets for CRISPR/Cas9-mediated modification. The first region of 18 bp included the VRN box sequence (**Supplementary Figure S1**). The second region is a 59 bp conserved fragment whose modification in common wheat varieties has not been previously described (**Supplementary Figure S1**) but has been reported for some spring accessions of *T. timopheevii, T. dicoccum*, and *T. compactum*. According to the algorithm of CRISPR/Cas9 target sequence design (20 bp upstream of the NGG PAM motif), we found five potential targets in the fragment containing the VRN box; nine sites were discovered between nucleotide positions −62 and −121 bp of the promoter (**Supplementary Figure S2** and **Supplementary Table S2**).

To eliminate low-potential targets, we tested the ability of predicted sgRNAs to guide the Cas9 protein to cleave the target sequences *via* an *in vitro* cleavage assay. It turned out that the designed ribonucleoprotein complexes corresponding to the target sequences of the VRN box have a very low ability to cleave the DNA template (0-10%). The rather high cleavage efficiency (equal to or higher than 50%) was demonstrated by three synthesized sgRNA-Cas9 complexes corresponding to sequences vr-30, vr-31, and vr-34 located in the second target region of the promoter (**Figure 1A** and **Supplementary Figure S3**). The sgRNAs mentioned above as being the most active were used to create genetic vectors to produce wheat plants with novel mutations. Each target sequence of vr-30, vr-31, or vr-34 was synthesized and fused to the default sgRNA scaffold under the control of the *U6* promoter (**Figure 1B**) in the pU6-gRNA vector (Shan et al., 2013).

### Generation of primary wheat plants carrying indels in the *vrn-A1* promoter by CRISPR/Cas9-mediated gene editing

Three uniform plasmids carrying one of the sgRNAs were independently co-transformed with Cas9-expression cassette pGCB (**Figure 1B**) into ‘Chinese Spring’ embryogenic calli using a particle inflow gun. Following the dual selection protocol (Miroshnichenko et al., 2020) we obtained 23 independent T0 transgenic plants carrying both the Cas9 gene and the sgRNA sequence (**Supplementary Table S3** and **Supplementary Figure S4**). All Cas9+sgRNA positive plants, including nine T0 plants carrying vr-30, eight T0 plants carrying vr-31, and six T0 plants carrying vr-34, were screened for indels. The target region of the *vrn-A1* promoter was amplified from the genomic DNA of T0 transgenic plants, and the PCR products were Sanger-sequenced to detect possible alterations. According to the analysis, independent events transformed with vr-30 or vr-34 sgRNA did not carry any modification of the target sequence. In contrast, the DNA sequencing charts of independent transgenic events transformed with pgRNA-VRNA#31 exhibited new peaks of bases, specifically 3–4 bp upstream of the PAM site (**Supplementary Figure S5**). In total, we obtained 4 primary mutant plants from 603 explants, and the efficiency of CRISPR/Cas9 editing was 0.7% (**Supplementary Table S3**).

**Figure 2:**
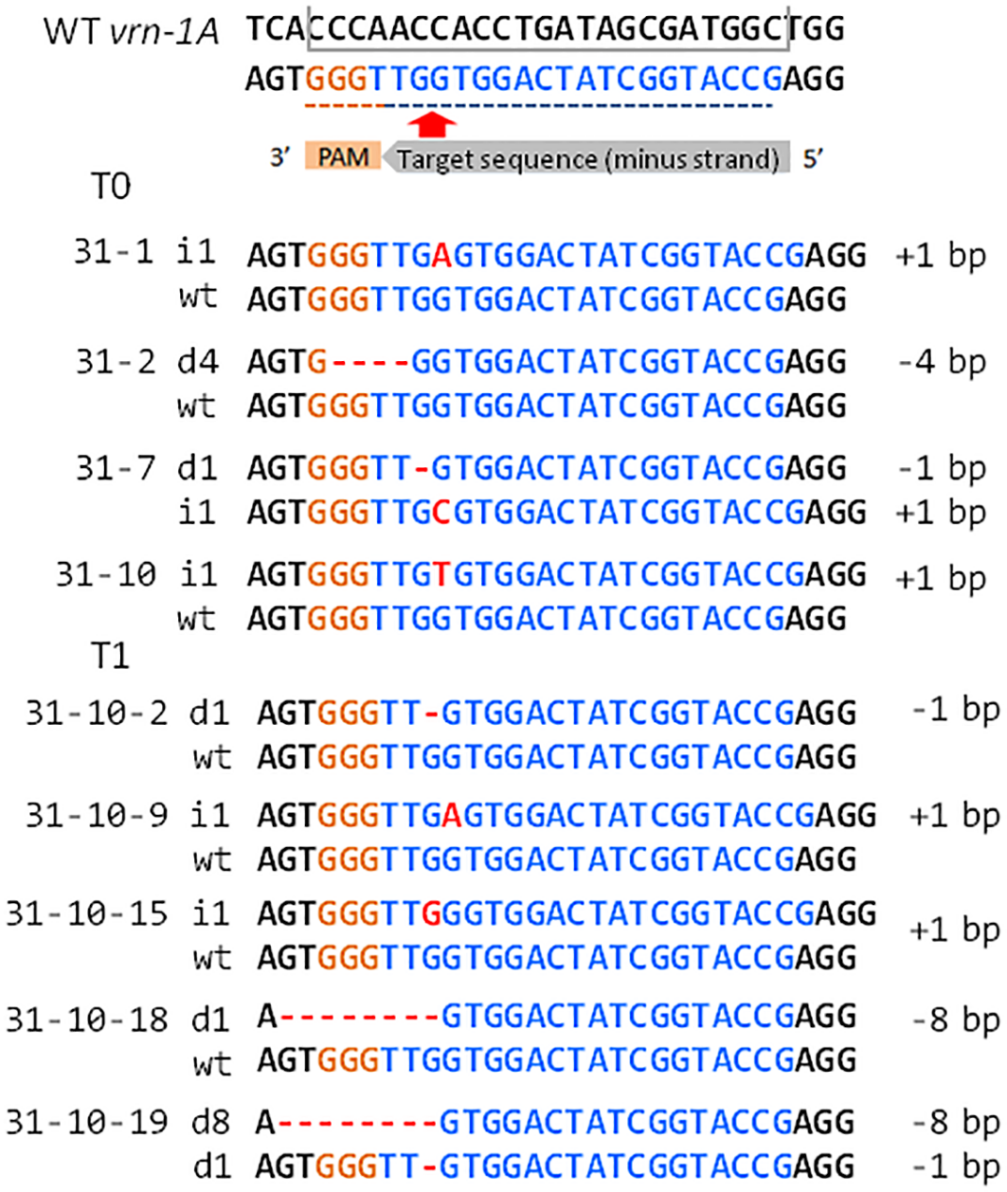
Various types of CRISPR/Cas9-induced mutations detected by amplicon sequencing in T0 and T1 wheat plants transformed with pgRNA-VRNA1#31. Bases in the black rectangle represent the target sequence vr-31, orange bases indicate the protospacer adjacent motif (PAM), and blue bases show the guide RNA-associated region, with sequences in red corresponding to insertion or deletion of bases.

Alignment of the PCR amplicons against the reference DNA sequence showed that two plants were monoallelic mutants, with each carrying a 1 bp insertion, and one plant was a biallelic mutant with a 1 bp deletion and a 1 bp insertion (**Figure 2**). Another mutated plant demonstrated monoallelic disruption of the NGG-PAM sequence due to the deletion of 4 nucleotides. The algorithm that we used to find targets for introducing mutations allows the selection of the most appropriate sequences, minimizing the off-target effects of CRISPR/Cas9 manipulations (Doench et al., 2016). A BLAST search of the reference genome sequence (genome assembly release 6.05.21) of the ‘Chinese Spring’ wheat cultivar (IWGSC CS RefSeq v2.1, GCF_018294505.1) showed that there were no perfectly matched targets with complete similarity to the vr-31 sgRNA sequence in wheat. Only one potential non-target site with a single nucleotide mismatch was found. It was located in the promoter sequence of the homeologous *vrn-B1* gene. Sanger sequencing of the corresponding amplicons produced from the B subgenome of four mutated plants #1, #2, #7, and #10 showed no changes in the promoter region of homeologous *vrn-B1* gene (**Supplementary Figure S6**). To confirm the specificity of *vrn-A1* editing, the promoter region of the *Vrn-D1* gene, which bears some similarity to the vr-31 sgRNA sequence, was also sequenced, but no off-target events were found (**Supplementary Figure S6**).

### Inheritance and segregation of induced mutations in subsequent generations and generation of homozygous transgene-free wheat lines carrying the novel alleles

Inheritance of mutant alleles was further investigated in M1 plants through the production of homozygous transgene-free populations for heading time evaluation. The segregation of transgenes was tracked based on a PCR assay for the presence of *GFP* (element of pGCB) and sgRNA scaffold region (element of pgRNA-VRN#31) sequences, and only plants lacking both transgenic sequences were accepted as being transgene-free. The proportion of transgene-free plants ranged from 16% to 78% in the progeny of three primary mutated events (**Supplementary Figures S8–S10**). Due to the limited number of seeds produced by the 31-1 primary plant, it was not possible to identify transgene-free progeny, as all germinated T1 plants inherited the Cas9 sequence (**Supplementary Figure S7** and **Supplementary Table S4**).

The mutations detected in 31-1, 31-2, and 31-7 primary plants were transmitted to the T1 generation without the occurrence of additional editing. The expected segregation pattern for monoallelic and biallelic mutations detected in 31-2 and 31-7 primary plants easily conforms to Mendelian distribution (1 (homozygous for mutation 1):2 (biallelic):1 (WT or homozygous for mutation 2)) (**Supplementary Figures S8, S9** and **Supplementary Table S4**). The segregation ratio of mutations in T1 plants of 31-1 did not follow the pattern for Mendelian inheritance (**Supplementary Figure S7**; **Supplementary Table S4**), and this could be explained by the limiting number of available T1 seeds produced by the primary plant. At the same time, the occurrence of additional editing was found in the progeny of 31-10 primary plant. Overall, five additional mono- and biallelic indel variants were discovered (**Supplementary Figure S10**). Aside from the 1 bp insertion that arose in 31-10 primary plant, two new types of 1 bp insertions, a 1 bp deletion and an 8 bp deletion, were detected in the *vrn-A1* promoter of T1 plants (**Figure 2**). All new modifications are proximal to the PAM site of vr-31 sgRNA. We performed Sanger sequencing analysis of some progeny of the self-pollinated T1 plants (including transgenic individuals carrying WT allele) and found no additional edits in the T2 generation (**Supplementary Table S5**). Indel mutations discovered in the T1 plants of 31-10 were easily heritable, and T2 plants carrying homozygous alleles of the additional edits were successfully detected (**Supplementary Table S5**).

In total, after sequencing the T1 and T2 progenies of four primary events, we detected seven CRISPR/Cas9-edited homozygous alleles of *vrn-A1* promoter (**Supplementary Table S6**). Some of the new edits, such as the 1 bp deletion, were generated as a result of independent editing. Tracking of the segregation of transgenes encoding *BAR*, *GFP*, *Cas9*, and sgRNA resulted in the acquirement of six GE populations (M3-M4 progenies) carrying CRISPR/Cas9-edited homozygous alleles without transgenic inserts (**Supplementary Figure S11**). In parallel, the selection of several transgene-free null-segregants was performed through genetic segregation of various GE monoallelic plants for use in comparative analysis.

### Characterization of the growth habit of genome-edited wheat lines

To test the influence of the introduced indels on the heading time, we planted nine GE populations (**Supplementary Table S5**) in a climate-controlled greenhouse (day/night temperature of 22-25 °C/18-20 °C with 16/8 h photoperiod) together with corresponding null-segregants derived from the same primary event (if available) for comparison with nontransformed ‘Chinese Spring’ plants. Plants were cultivated without vernalization treatment. Starting from the 58th day after germination, the first emergence of heads was observed in individual plants, whereas the peak of heading occurred at 63-66 days. In this experiment, the average heading time of ‘Chinese Spring’ plants was 63.8 days (n=61). A similar timing of first head appearance (63.6 days, n=45) was also observed in nontransgenic WT31-10 plants that had not inherited induced mutations and represent the seed generation of the primary 31-10 plant. The null-segregants WT31-2 (the nontransgenic siblings of the 31-2 primary plant) showed somewhat later heading at 65.3 days (n=27); however, the observed difference (CS vs. WT31-2) was not significant according to the ANOVA test (*p*=0.2214) (**Supplementary Table S7**).

**Figure 3:**
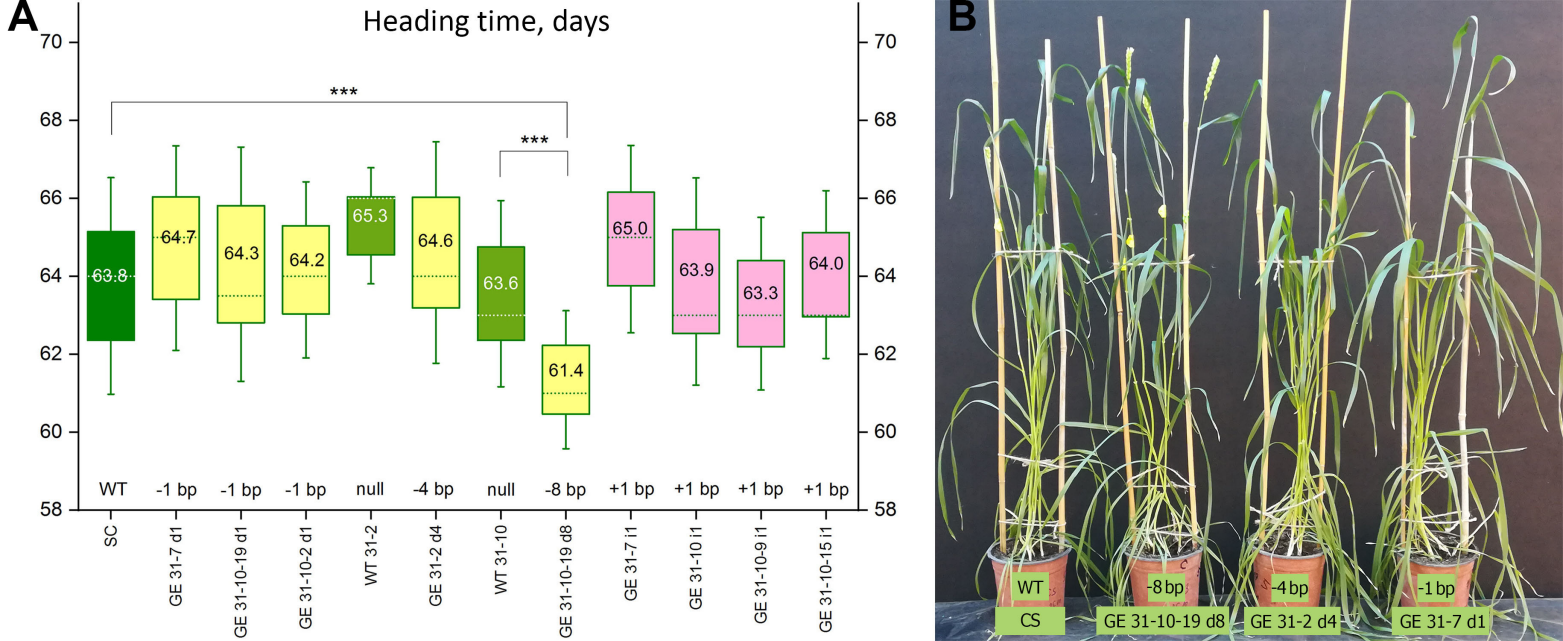
Characterization of the growth habit of genome-edited wheat lines. **(A)** Heading dates of the plants carrying various types of CRISPR/Cas9-induced homozygous mutations in the target region of *vrn-A1* promoter; plants carrying the wild-type allele of *vrn-A1* (‘Chinese Spring’ and two null siblings) are indicated in green; GE lines with base pair deletions are shown in yellow, GE lines with base pair insertions are shown in pink. Heading time values were compared through ordinary one-way ANOVA test, and significant differences between the lines are shown according to Turkey’s multiple range test (***p < 0.001). **(B)** The growth habit of the plants carrying the wild-type allele of *vrn-A1* (WT, ‘Chinese Spring’) and three lines carrying homozygous alleles of *vrn-A1* with various types of nucleotide deletions. The experiment was performed without vernalization treatment in glass greenhouse at 16-18°C/19-22°C (night/day) under a long photoperiod (16 h light).

No clear differences in heading dates were observed between ‘Chinese Spring’ and the group of GE lines carrying different single nucleotide insertions (**Figure 3A**). The average time for the first spike appearance was 65.0 days for GE31-7 i1 (n=45), 64.0 days for the GE31-10 i1 (n=45), 63.9 days for the GE31-10-9 i1 (n=45), and 63.3 days for the GE31-10-15 i1 (n=30). Since the three last lines resulted from the genome editing of the chimeric T0 event 31-10, it was possible to compare the resulting heading dates with the null-segregant WT31-10. Statistical analysis showed no significant difference when compared with either ‘Chinese Spring’ (p=0.9996 for CS vs. GE31-10-9 i1; *p*>0.9999 for CS vs. GE31-10-7 i1; *p*> 0.9999 for CS vs. GE31-10-15 i1) or WT31-10 plants (*p*=0.9972 for WT31-10 vs. GE31-10-9 i1; *p*>0.9999 for WT31-10 vs. GE31-10-7 i1, *p*=0.9986 for WT31-10 vs. GE31-10-15 i1). Since the primary T0 plant 31-7 is biallelic, it was not possible to compare the heading time of GE 31-7 i1 with an appropriate null sibling, whereas the deviation from ‘Chinese Spring’ was not confirmed (*p*=0.3494, CS vs. GE31-7 i1).

The average heading time for the group of GE lines carrying the same deletion of a single nucleotide in the target region of the *vrn-A1* promoter varied from 64.7 days in GE 31-7 d1 (homozygous progeny of the primary plant 31-7) to 64.3/64.2 days in GE31-10-19 d1/GE31-10-2 d1 (progeny of chimeric event 31-10). Since the average number of days to head emergence in these GE lines was similar to that of ‘Chinese Spring’, no statistical significance was found (**Supplementary Table S6**). Similarly, the comparison between heading dates of GE31-10-19 d1/GE31-10-2 d1 lines and WT31-10 (null siblings of the same primary event) did not reveal any significant differences (**Figure 3**).

The absence of four nucleotides in the promoter region of the *vrn-A1* gene in the GE31-2 d4 line also had no effect on the heading time (**Figure 3**). Although the GE31-2 d4 plants headed relatively later (64.6 days) than the wild-type ‘Chinese Spring’ plants (63.8 days) but slightly earlier than the null sibling plants of WT31-2 (65.3 days) originated from the same primary event, the observed deviations were within the experimental error (p=0.9895, WT31-2 vs. GE31-2 d4; *p*=0.7648, CS vs. GE31-2 d4).

Contrary to the six CRISPR/Cas9-induced mutations listed above, the loss of 8 nucleotides between −125 and −117 bp of the recessive *vrn-A1* promoter affected the heading/flowering time of wheat (**Figures 3A, B**). Plants of the GE31-10-19 d8 line headed, on average, 2.3-2.5 days earlier than ‘Chinese Spring’ and the corresponding null sibling WT31-10. Moreover, significant differences with a high level of confidence (*p*-value < 0.0001) were also found between the average head emergence date of the GE31-10-19 d8 line and the heading dates of most other mutated lines (**Supplementary Table S7**).

**Figure 4:**
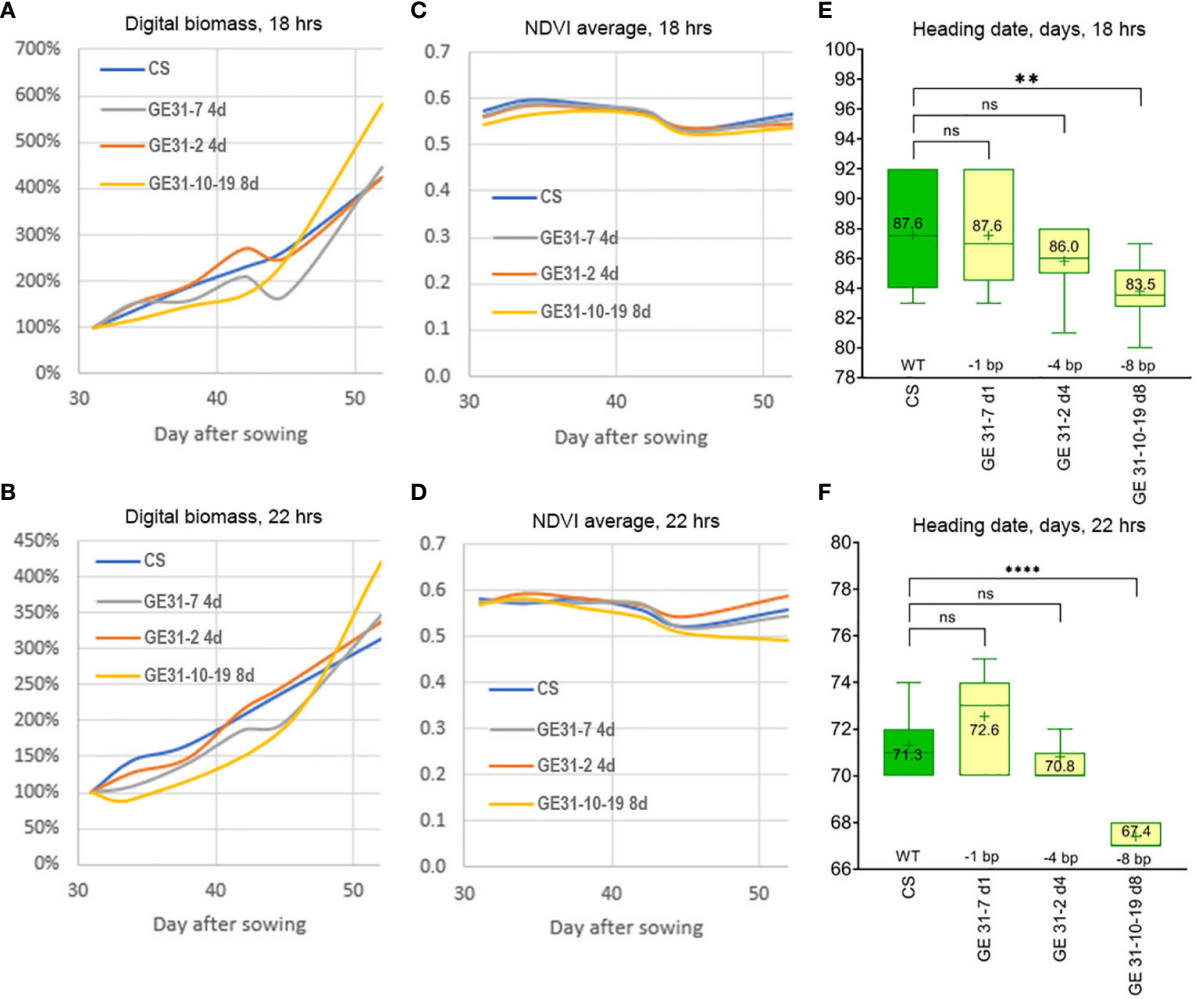
Evaluation of the growth habit of genome-edited wheat lines using growth chamber under two environmental regimes: **(A, C, E)** 18 h photoperiod at 22 ± 0.5°C; **(B, D, F)** 22 h photoperiod at 17 ± 0.5°C. A 3D model of wheat pots painted with NDVI-compliant pseudocolors. Changes in average values of **(A, B)** digital biomass and **(C, D)** NDVI of wheat plants between 30 and 50 days after sowing, determined with Trait Finder digital phenotyping system. Heading time of wheat lines under an **(E)** 18 h and **(F)** 22 h photoperiod; significant differences between the lines are shown according to Turkey’s multiple range test (**p < 0.005; ****p < 0.0005; ns: nonsignificant). .

When the wheat lines carrying various nucleotide deletions were cultivated in climate chambers under increased day length of 18 h (at 22 °C) or 22 h (at 17 °C), the GE31-10-19 d8 line also demonstrated earlier heading than plants of ‘Chinese Spring’ and GE lines GE31-7 d1 and GE31-2 d4 carrying 1 or 4 bp deletions (**Figure 4**). Despite the fact that digital phenotyping revealed more active increases in plant biomass under a 22 h photoperiod growth regime, no significant differences in the morphological stages of plant development between the GE lines and ‘Chinese Spring’ were found between the different day/night/temperature regimes (**Figures 4A, B**). The spectral NDVI index of GE lines and ‘Chinese Spring’ was similar throughout the period of automatic digital phenotyping until the tilling stage, when the NDVI index value of GE31-10-19 d8 started to decrease, especially at 22 h photoperiod (**Figures 4C, D**), whereas its digital plant biomass still showed a tendency to increase (**Figures 4B, C**). Plants of GE31-10-19 d8 reached the tillering stage 2-5 days earlier than the GE lines with minor nucleotide deletions and wild-type ‘Chinese Spring’. As a result, at 18 h photoperiod, the average heading time in plants of GE31-10-19 d8 was 83.5 days in contrast to 86.0-87.6 days for GE31-7 d1, GE31-2 d4, and ‘Chinese Spring’ plants (**Figure 4E**). Under the 22 h photoperiod regime (**Figure 4F**), the difference also was evident, as the average heading date of 67.4 days in GE31-10-19 d8 was shorter than 71.3 days in ‘Chinese Spring’ (*p* < 0.0001), while the differences between ‘Chinese Spring’ and two other lines were not statistically significant (*p*=0.1454, CS vs. GE31-7 d1; *p*=0.8251, CS vs. GE31-2 d4).

**Figure 5:**
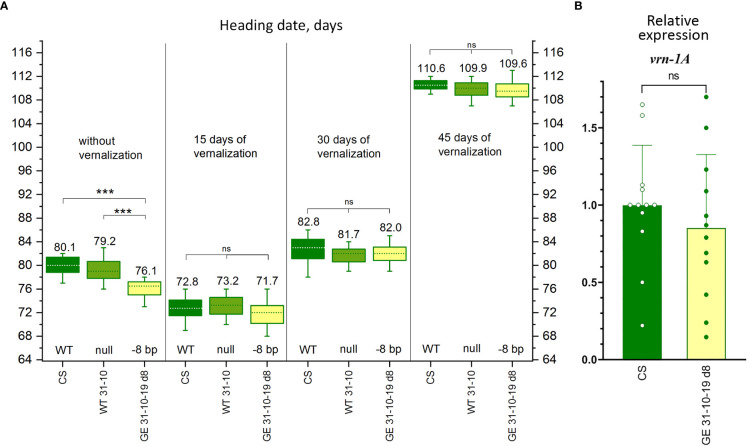
Characterization of the genome-edited wheat line carrying the 8 bp deletion in the promoter region of *vrn-A1.* **(A)** Effect of vernalization on the heading date of wheat plants carrying the 8 bp deletion (GE31-10-19 d8) in comparison with the ‘Chinese Spring’ plants and transgene-free null-segregant plants (WT31-10). NS: nonsignificant. **(B)** Relative expression of the *vrn-1A* gene in wheat plants carrying the 8 bp deletion in the promoter region of *vrn-A1* (GE31-10-19 d8) in comparison with the ‘Chinese Spring’ (CS) plants; Tukey’s multiple comparisons test, ***p < 0.001; ns: nonsignificant.

To further assess the relationship between the changes in growth habit and the CRISPR/Cas9-induced 8 bp deletion in the promoter region of *vrn-A1* gene, we compared the heading dates of unvernalized GE31-10-19 d8 plants with that of plants vernalized for 15, 30, and 45 days. Even the short vernalization of 15 days advanced the head emergence in such a way that all accessions, including GE31-10-19 d8, nontransgenic null sibling WT31-10, and ‘Chinese Spring’ headed earlier than the corresponding unvernalized plants (**Figure 5A**). Regardless of the duration of cold treatment (15, 30, or 45 days), the time to plant heading in GE31-10-19 d8 was similar to that of both ‘Chinese Spring’ and the null sibling WT control. In parallel, the unvernalized GE31-10-19 d8 plants headed, on average, 3-4 days earlier than the unvernalized ‘Chinese Spring’ or nontransgenic null sibling (**Figure 5A**).

To investigate the effect of mutations on the relative expression of the *vrn-1* gene, we assessed the expression levels in unvernalized plants at the early tilling stage in the fifth fully expanded leaf. The quantitative gene expression results showed no significant changes in the *vrn-A1* gene expression levels in CRISPR/Cas9-mutated plants carrying the 8 nt deletions compared with WT plants (**Figure 5B**).

Analysis of the harvested seeds showed that the presence of a 8 bp deletion in the *vrn-A1* promoter does not affect significantly the seed quality (**Supplementary Figure S12**). The thousand grain weight of GE31-10-19 d8 line (34.5 g) did not significantly differ from that of the nontransgenic null sibling (33.1) and wild-type ‘Chinese Spring’ (35.9 g) plants (**Supplementary Figure S12**). Although some increase in the total protein content of the grains was observed between the GE31-10-19 d8 line (18.7%) and wild-type ‘Chinese Spring’ (18.1%), the nontransgenic null sibling showed nearly the same average value (18.6%) as the plants carrying the new mutated *VRN-A1* allele. Similarly, no significant differences were observed across the GE line and two controls (48.4-49.1%) when the total starch content was analyzed (**Supplementary Figure S12**).

## Discussion

The effect of vernalization on plant flowering has been the subject of intensive investigation for decades. A number of key genes regulating the vernalization-related response have been identified in plants, especially in *Arabidopsis*, for which mutants or transgenic plants are easier to acquire (Kennedy and Geuten, 2020). In wheat, less information is available since the search and analysis of a natural mutant collection is complicated by polyploidy, high heterozygosity, and haplotype variation. Wheat is a transformation-recalcitrant species, and transgenesis is not as effective an approach since the functional activity of vernalization-related homeologs is largely dependent on noncoding sequences, such as introns and promoters. Here, the CRISPR/Cas9 approach was used to produce new mutant alleles and study the effect of mutation introduced into the promoter of the *VRN-A1* gene that is known to play a central role in regulating the response to vernalization and spring/whiter growth habit (Nishiura et al., 2014; Sharma et al., 2020).

In the present study, new mutations were successfully induced within 125-117 bp upstream of the start codon of the recessive *vrn-A1* gene using one of the designed gsRNAs. The targeted mutations were induced by the expression of CRISPR/Cas9-editing vectors and generally showed a standard germline transmission pattern that resulted in the generation of stable homozygous mutant populations free of transgenic inserts. Although the use of the CRISPR/Cas9 system in our study was relatively successful with regard to generating GE wheat plants, there were some issues still requiring additional attention. One such issue was the appearance of new indels in the progeny of the primary edited 31-10 plant. It has been previously reported that heterozygous CRISPR/Cas9-edited plants may sometimes experience additional mutations in subsequent generations when they still carry both functionally active Cas9/sgRNA cassettes and the WT allele of the target sequence (Wang et al., 2018). No new types of mutation were detected when analyzing individual monoallelic T2-T3 progenies of the 31-10 plant, indicating that the 31-10 primary plant is chimeric, and the modification of nucleotide sequences occurred independently in various cells after the delivery of Cas9 and sgRNA vectors. Since chimeric GE plants are rarely found in cereals, it can be assumed that their appearance is associated with features of the genotype, CRISPR/Cas9 encoding vectors, or the transformation protocol. In any case, our observations indicate that the chimerism of generated GE events cannot be ruled out and is one of the issues associated with the use of a biolistic approach to producing new mutations in wheat.

In this study, we set out to investigate whether the modification of specific promoter sequence of the recessive *vrn-A1* gene may influence the growth habit of the semi-winter variety ‘Chinese Spring’. In total, seven independent target-specific mutations were identified in the genome of wheat plants between 125 and 117 bp upstream of the start codon of *vrn-A1* gene. Mutations in this part of the promoter have not previously been studied in bread wheat, as natural mutations in accessions of *T. aestivum L*. have never been reported. The most studied “critical region” of *VRN-A1* promoter, namely the VRN box, is well known to influence the spring/winter growth habit of wheat, and it was initially included in our study. Due to the limited number of possible CRISPR/Cas9 targets in the 16 bp sequence and the low cleavage efficiency of the designed gsRNAs, we failed to artificially induce new mutations in the VRN box. This is another issue encountered in our study that is associated with the application of the CRISPR/Cas9-based approach to a hexaploid species for precise genome editing.

CRISPR/Cas9-mediated editing has already been used to induce mutations in flowering time-related genes in *Arabidopsis*, soybean, and wheat, but these frameshift mutations aimed to inactivate the functionality of the *AtFT* (Hyun et al., 2015), *GmFT2* (Cai et al., 2018), and *Taft-d1* (Chen et al., 2022) genes. In our study, the induction of mutations was not aimed at knocking out the *VRN-A1* gene but, rather, at changing its functional activity. Induction of a minor change, such as 1–4 nt indels between −121 and −117 bp, had no evident effect on head emergence. Heading time was affected only when there was a longer deletion of 8 bp between −125 and −117 bp. It is encouraging that no negative effects on the phenotype due to the presence of such mutations were observed. Nonvernalized homozygous GE31-10-19 d8 plants carrying the new mutated *vrn-A1* allele did not demonstrate phenotypic differences from ‘Chinese Spring’ according to digital phenotyping, at least until the tilling stage. Moreover, there were no clear differences in yield characteristics, such as the thousand grain weight and the contents of total protein and starch in kernels.

The effect of the absence of 8 bp on the date of heading was steadily manifested under unvernalized conditions at various long day/temperature regimes. For all long day cultivations, including 16/8, 18/6, and 22/2 (day/night, h) growth regimes, GE31-10-19 d8 plants demonstrated the earlier appearance of ears. When plants of mutated lines with deletion of 1, 4, or 8 bp and ‘Chinese Spring’ were cultivated at a temperature of 22°C, they demonstrated a nearly two-week delay in earing/flowering compared with cultivation at 17°C. These data are in agreement with observations of Kiss et al. (2017), who found a link between lower expression levels of *VRN-1* and *VRN-3* at higher temperatures (25°C < 18°C < 11°C) and delayed apex/plant developmental phases, especially under long day photoperiods. Despite variation in the rate of development observed in our study, the plants of the mutant line carrying the 8 bp deletion demonstrated, on average, 2-4 days earlier heading than the lines with minor mutations (1 or 4 bp deletions) and WT under both warmer (22°C) and cooler (17°C) temperature conditions.

When GE31-10-19 d8 plants with mutated *vrn-A1* promoter were exposed to low temperatures of 5 ± 1°C, even for a period of two weeks, the effect was no longer observed. Based on the accepted hypothesis of a functioning *VRN* gene network (Distelfeld et al., 2009; Nishiura et al., 2014), it can be assumed that vernalization stimulates the expression of recessive *vrn-A1* in wheat, and such an increase apparently overlaps the expression of *VRN-1* homologs observed in the mutant line without exposure to low temperature. This indicates that the deletion of 8 bp between −125 and −117 bp of the recessive *vrn-A1* promoter is effective only when plants do not undergo vernalization.

When we examined the changes in *vrn-A1* expression levels in the unvernalized mutated GE31-10-19 d8 line at the early tillering stage, we could not find statistically significant differences when comparing with ‘Chinese Spring’ plants (**Figure 5B**). The unchanged *vrn-A1* expression levels make it difficult to interpret the acceleration of ear appearance, and we suppose that the 8 bp mutation may directly or indirectly affect the functioning of various *VRN-1*-linked genes, which may include but are not limited to such genetic pathways as involving the circadian clock, the nutritional and developmental status of the plant, and a variety of biotic and abiotic signals. The analysis using PLACE and PlantCARE databases revealed that the 8 bp deletion disrupts cis-acting elements such as WRE3 (CCACCT) and MYBPZM (CCWACC) located in the native *vrn-A1* promoter at positions −117 and −121 bp, respectively, relative to the translation initiation site. WRE3 is known as a stress-responsive element for wounding and pathogen attack, whereas MYBPZM is the core of the consensus binding site (MYB homolog) of the maize P gene that determines the red pigmentation of flower organs by direct activation of genes that affect flavonoid biosynthesis (Grotewold et al., 1994). Since the transition to the heading stage in wheat is influenced by a whole range of different factors, not only due to the presence of cold- of light-responsive cis elements, it is possible that the loss of WRE3 and MYBPZM cis elements modified the fine regulation of *vrn-A1* promoter activity that, in turn, may contribute to the alterations observed in the heading time of the GE31-10-19 d8 line. Regardless, the relationship between the structural and functional differences of newly produced mutation should be further explored and validated. Numerous studies have revealed a highly changeable level of *VRN-A1* expression at different developmental stages of wheat plants (Loukoianov et al., 2005; Basualdo et al., 2015). Taking into account the rather minor changes in the heading date, a more extensive study involving a higher number of plants at various stages of plant development should be carried out, in the future, to clarify the relationship between the CRISPR/Cas9-induced 8 bp deletion and the expression levels of *vrn-A1* and other VRN-associated genes.

The results of our study, intending to expand the allelic variants of the *VRN-1* gene by CRISPR/Cas9-mediated induction of mutations, may contribute to uncovering the roles of various promoter loci in regulating the heading time of temperate cereals. Only 1 out of the 12 predicted gsRNAs was effective in inducing mutations in the *vrn-A1* promoter, but this problem of inefficacy can potentially be solved in the future by the application of other endonucleases to expand the number of PAM combinations and target sequences (Wang et al., 2021). A 2–3-day change in heading/flowering time, which was characteristic of the novel mutant plants carrying a homozygous 8 bp deletion in the *vrn-A1* locus between 125 and 117 bp upstream of the start codon, may be in demand in future wheat breeding. Such a trait provides an additional opportunity for fine-tuning flowering time in bread wheat designed for regions with a variable climate. However, confirmation of a phenotype in the field and crossbreeding with other varieties will be useful for determining whether the observed acceleration remains stable. Additional studies are still needed to identify the environmental conditions under which this trait will be maximized in mutant plants.

## Data availability statement

The data presented in the study are deposited in the Sequence Read Archive (SRA) repository, accession number PRJNA88662, link: https://www.ncbi.nlm.nih.gov/bioproject/?term=PRJNA886662.

## Author contributions

ES, GK, SD conceptualization; design of research and resources; DM, VT, AK, AP, TS, DL, LN, OS, MD performed the experiments; DM, VT, DL, MD analyzed the data, DM wrote the manuscript with input from all authors. All authors have read and agreed to the published version of the manuscript.

## Funding

This work and publication were supported by the Kurchatov Genomics Center of All-Russia Research Institute of Agricultural Biotechnology, agreement #075-15-2019-1667.

## Acknowledgments

The authors would like to thank the staff of the large-scale research facilities “FITOTRON” (registration number 2-2.9) of the Shemyakin-Ovchinnikov Institute of Bioorganic Chemistry for the growing of the donor and genome-edited wheat plants.

## Conflict of interest

The authors declare that the research was conducted in the absence of any commercial or financial relationships that could be construed as a potential conflict of interest.

## Publisher’s note

All claims expressed in this article are solely those of the authors and do not necessarily represent those of their affiliated organizations, or those of the publisher, the editors and the reviewers. Any product that may be evaluated in this article, or claim that may be made by its manufacturer, is not guaranteed or endorsed by the publisher.
