# Supplementary material for: CRISPR/Cas9-induced modification of the conservative promoter region of VRN-A1 alters the heading time of hexaploid bread wheat
Source: Front Plant Sci. 2022 Dec 5;13:1048695. doi: 10.3389/fpls.2022.1048695 (PMC9760837; doi:10.3389/fpls.2022.1048695)
Supplement: Supplementary file 1 [file DataSheet_1.pdf]

frontiers

Supplementary Material

CRISPR/Cas9-induced modification of the conservative promoter region of *VRN-A1* alters the heading time of hexaploid bread wheat

Dmitry Miroshnichenko, Vadim Timerbaev, Anna Klementyeva, Alexander Pushin, Tatiana Sidorova, Dmitry Litvinov, Lubov Nazarova, Olga Shulga, Mikhail Divashuk, Elena Salina, Gennady Karlov, Sergey Dolgov

TGAAAGGAAAAATTCTGCTCGTTTTTTTGCTCTGTGGTGTGTGTTTGTGGCGAGAGAA
AATGATTTGGGGAAAGCAAAATCCGGAGATTCGCACGTACGATCGTTCGACACGTCGA
CGCCCGGCGGGCCCGGGGTGGGGCATCGTGTGGCTGCAGGACCGCGGGGCCCCGCAA
AGCGGGCCGGGCCAATGGGTGCTCGACAGCGGCTATGCTCCAGACCAGCCCGGTATTG
CATACCGCGCTCGGGGCCAGATCCCTTTAAAAACCCCTCCCCCCCTGCCGGAATCCTC
GTTTTGGCCTGGCCATCCTCCCTCTCCTCCCCTCTCTTCCACCTCACGTCCTCACCCAA
CCACCTGATAGCCATGGCTCCGCCGCCTCGCCTCCGCCTGCGCCAGTCGGAGTAGCCG
TCGCGGTCTGCCGGTGTTGGAGGGTAGGGGCGTAGGGTTGGCCCGGTTCTCGAGCGG
AGATGGGGCGGGGGAAGGTGCAGCTGAAGCGGATCGAGAACAAGATCAACCGGCAG
GTGACCTTCTCCAAGCGCCGCTCGGGGCTTCTCAAGAAGGCGCACGAGATCTCCGTGC
TCTGCGACGCCGAGGTCGGCCTCATCATCTTCTCCACCAAGGGAAAGCTCTACGAGTT
CTCCACCGAGTCATGGTAAATTAAGCACGCGCTGTCTTTAAATTTGTTCCCCAATACGC
CTTCG

Supplementary Figure S1. The *vrn-A1* promoter sequence with target regions (outlined as boxes) for CRISPR/Cas9 genome modifications. Yellow letters mark the VRN box, green letters mark the 50-bp sequence located between –62 and –112 bp of the promoter region.

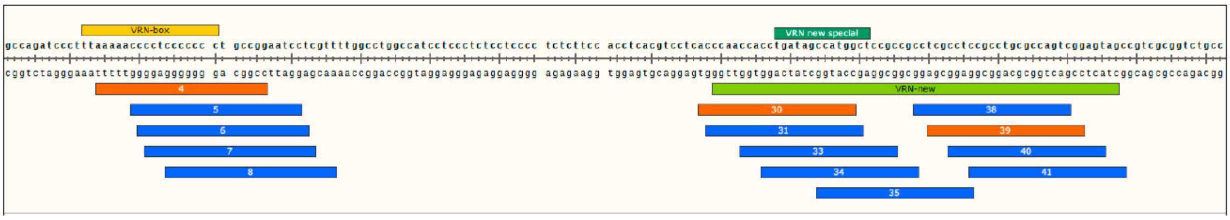

Supplementary Figure S2. Presentation of the recessive *vrn-A1* promoter sequence of 'Chinese Spring' (used as wild-type) and target locations for CRISPR/Cas9-mediated editing of two promoter regions, including thr VRN-box (highlighted in yellow) and the conserved 59 bp region (highlighted in green); positively oriented target sequences are highlighted in red, reverse oriented sequences are highlighted in blue.

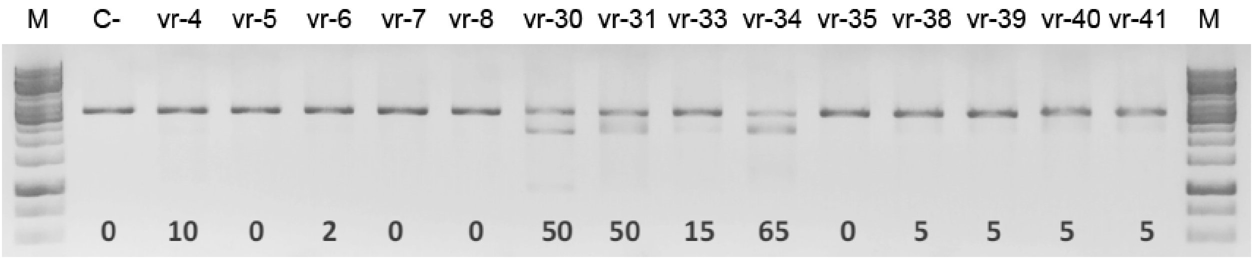

Supplementary Figure S3. In vitro cleavage assay of the *vrn-A1* promoter DNA fragment with sgRNA candidates. A linearized DNA substrate of 'Chinese Spring' was used as a negative control (WT), without addinion of the pre-assembled RNP complex. The band intensity of the non-cleaved WT was taken as 100% and densitometrically compared with the values of experimental bands; the cleavage efficiency (indicated in the lower part of the gel) was determined as the difference between WT (= 0% efficiency) and the value for each individual sgRNA.

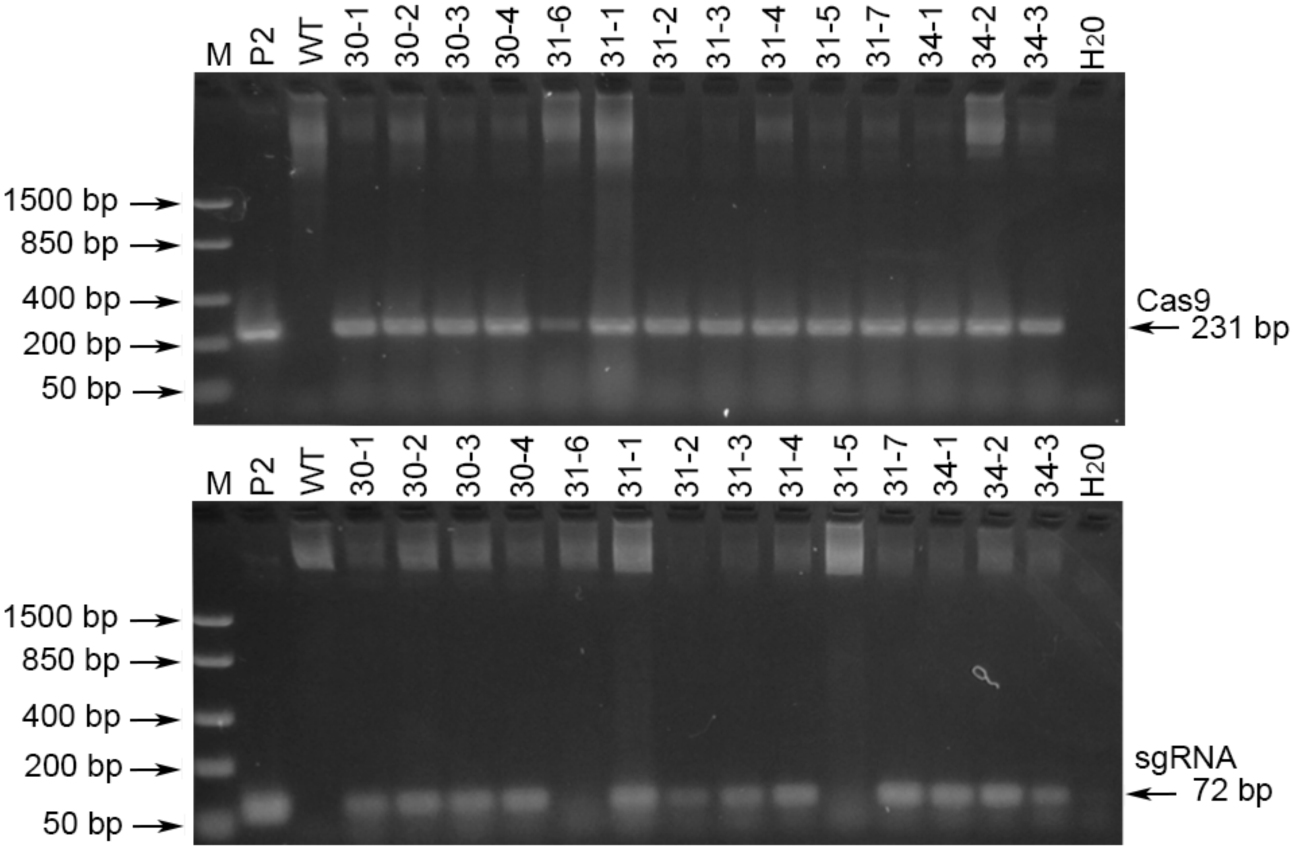

Supplementary Figure S4. Example identification of transgenic events. Putative transgenic wheat plants were analyzed for the presence of representative PCR products of Cas9 and sgRNA amplified from the DNA of individual events. Lane M, DNA ladder as molecular weight marker; lane P1, plasmid DNA of pGCB; lane P2, plasmid DNA of pgRNA-VRNA1#30; lane WT, untransformed 'Chinese Spring' wheat plant; numbers represent individual primary T0 plants, generated after transformation with pgRNA-VRNA1#30 (30-1, 30-2, 30-3, 30-4, 30-7), pgRNA-VRNA1#31 (31-1, 31-2, 31-3, 31-4, 31-5, 31-6, 31-7) and pgRNA-VRNA1#34(34-1, 34-2, 34-3)

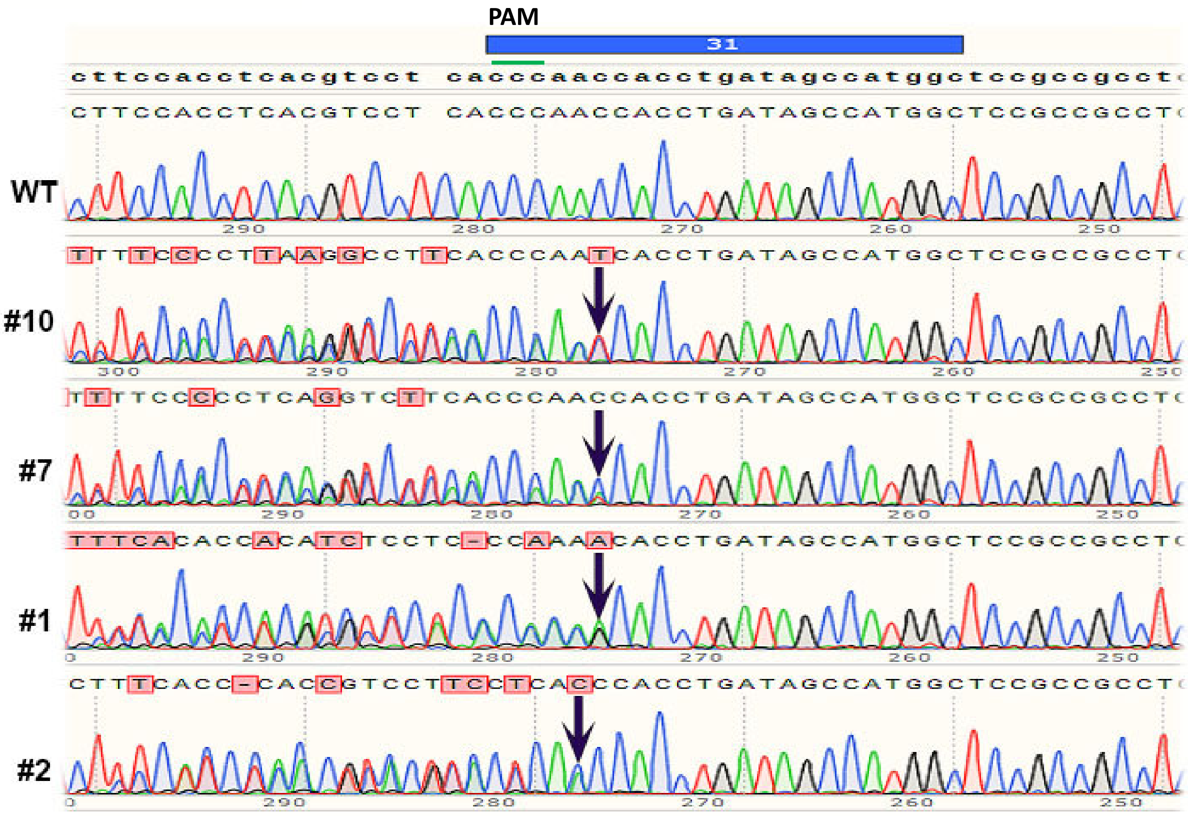

Supplementary Figure S5. Sequencing chromatogram of the PCR products demonstrating either loss of peak/gain of peak or overlapping peaks around the target site in the mutate lines: #1, #2, #7, and #10. Sequence of the recessive *vrn-A1* promoter (WT) is shown on top of the sequencing histogram for comparison.

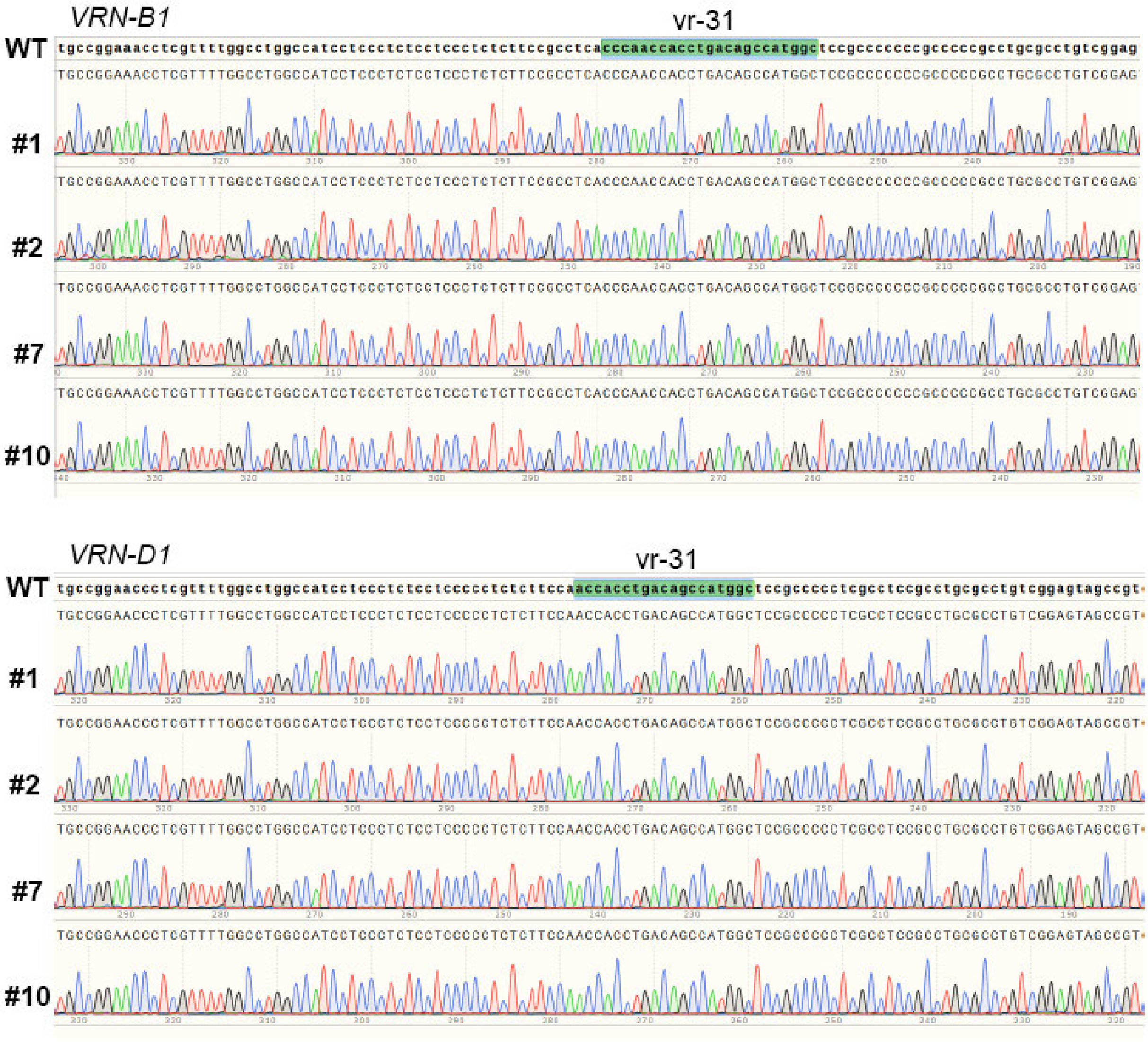

Supplementary Figure S6. Analysis of possible changes in the nucleotide sequence of the promoter region of the *vrn-1B* and *Vrn-1D* genes in transgenic wheat plants #1, #2, #7, and #10 carrying induced mutations in the *VRN-A1* sequence. Reference sequences of the promoter region of the *vrn-1B* and *Vrn-1D* genes are shown on top of sequencing histograms and includ the fragment that resembles the sequence of the vr-31 sgRNA.

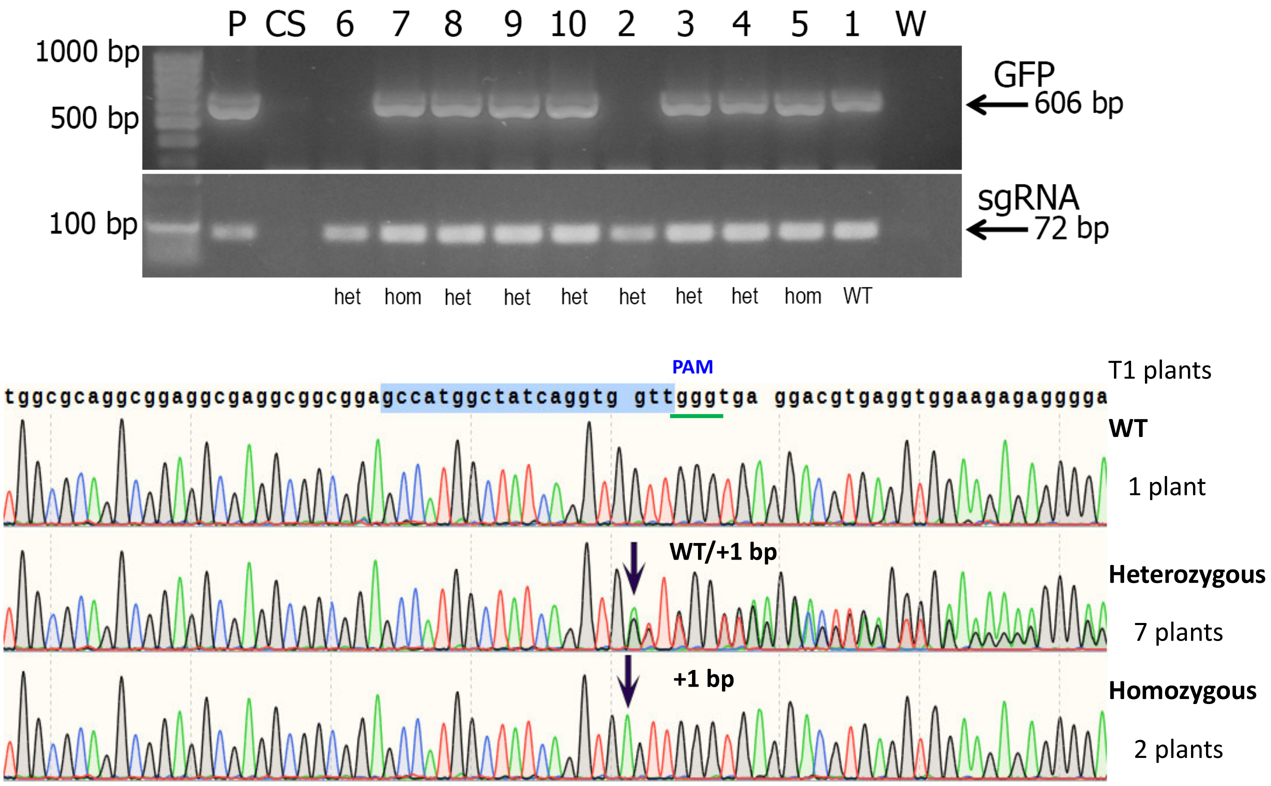

Supplementary Figure S7. Inheritance analysis of 31-1 primary event carrying a 1 bp monoallelic insertion in the target region of the *vrn-A1* promoter.

Top panel. PCR analysis of transgene segregation (GFP and sgRNA scaffold); lane P, the corresponding plasmid DNA of pGCB or pgRNA-VRNA1#31; lane CS, untransformed ‘Chinese Spring’ wheat plant; numbers 1-10 represent individual T1 plants, generated after selfing of the 31-1 primary plant, with the indication of mutation inheritance type (WT, heterozygous, homozygous) in the bottom of panel.

Bottom panel. The typical chromatograms of T1 plants after Sanger sequencing of the *vrn-A1* promoter, including null-segregants not inheriting mutations (WT), heterozygous plants showing overlaid signals (similar to the parent T0 plant), and homozygous plants demonstrating the uniform additional peak in both alleles; PAM sequences are marked in green.

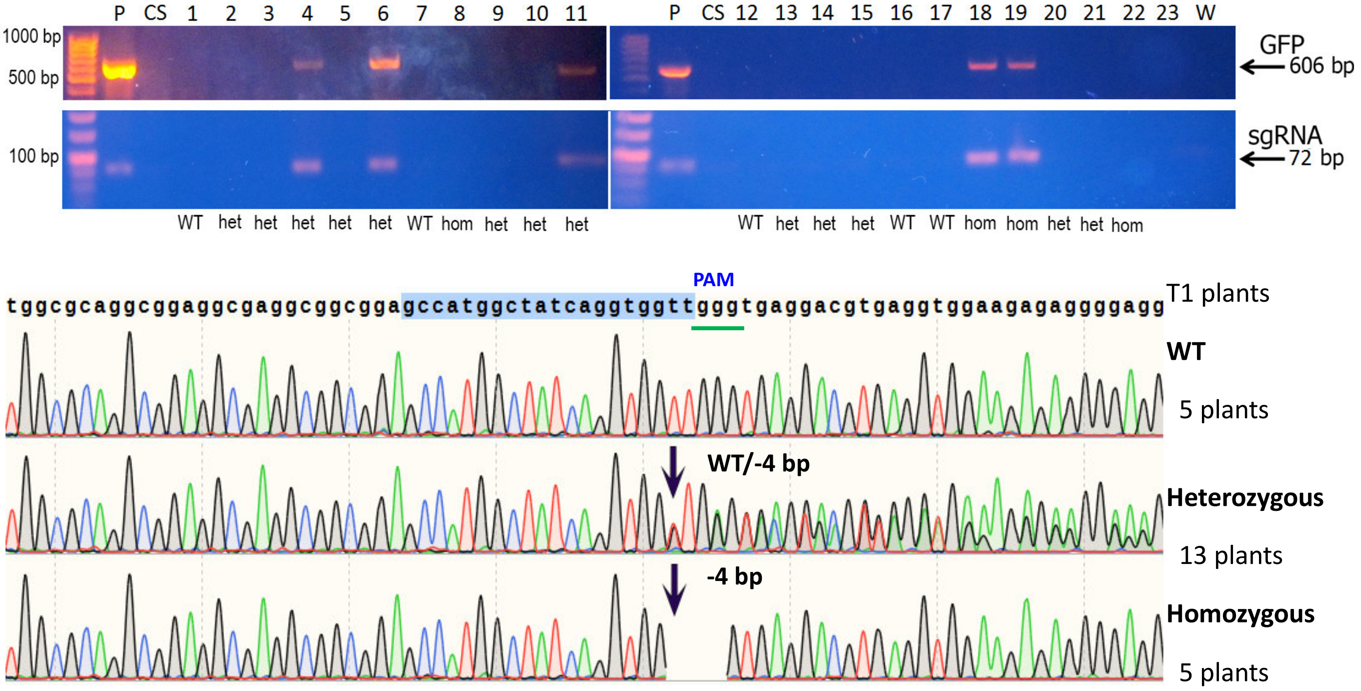

Supplementary Figure S8. Inheritance analysis of 31-2 primary event, carrying the monoallelic deletion of four nucleotides in the target region of the *vrn-A1* promoter.

Top panel. PCR analysis of transgene segregation (GFP and sgRNA scaffold); lane P, the corresponding plasmid DNA of pGCB or pgRNA-VRNA1#31; lane CS, untransformed 'Chinese Spring' wheat plant; numbers 1-23 represent individual T1 plants generated after selfing of the 31-2 primary plant, with the indication of mutation inheritance type (WT, heterozygous, homozygous) in the bottom of panel.

Bottom panel. Sequencing chromatograms of the *vrn-A1* promoter fragment demonstrating new combinations of CISPR/Cas-9-induced indels in various T1 plants; T1 plants PAM sequences are marked in green.

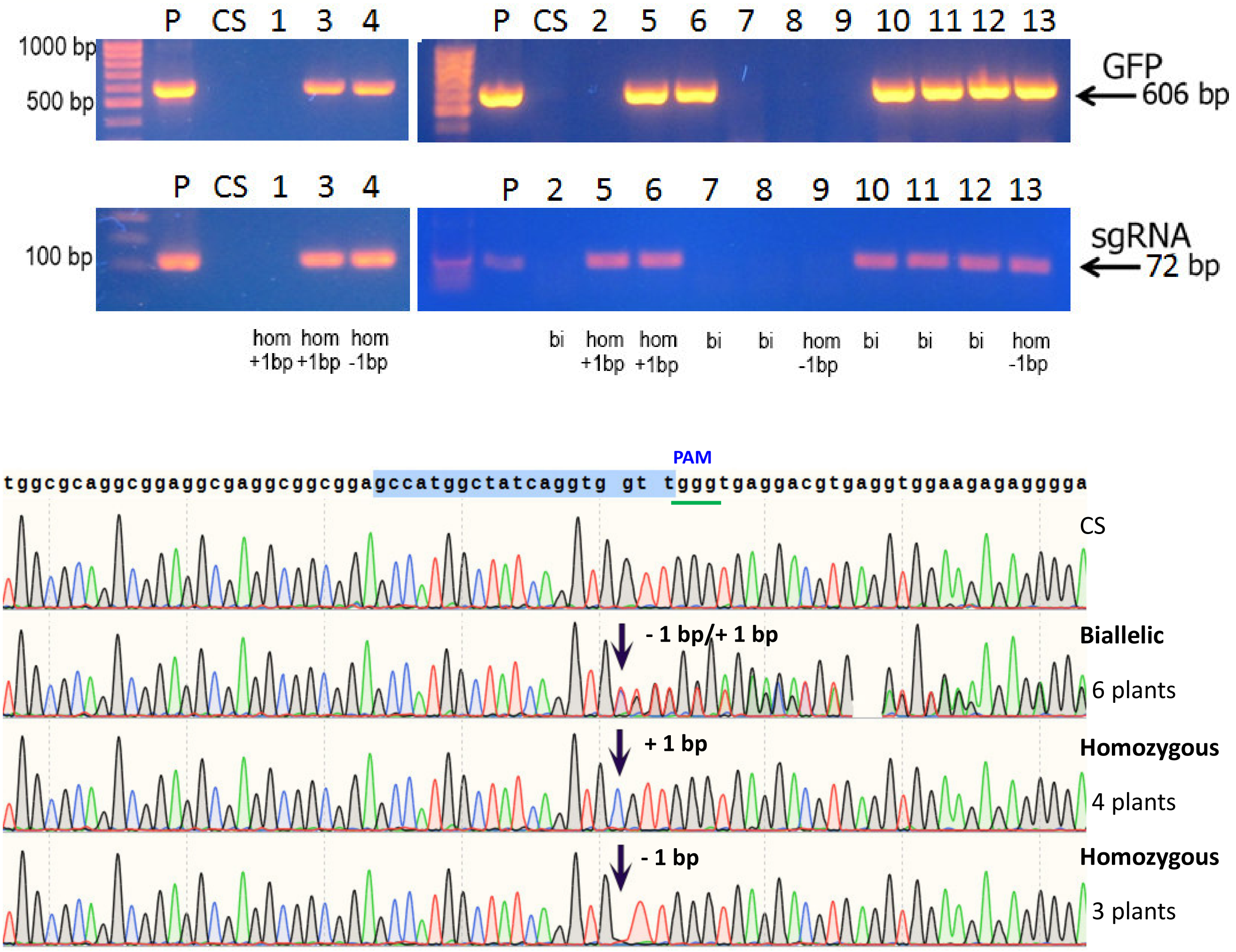

Supplementary Figure S9. Inheritance analysis of the 31-7 primary event carrying the biallelic nucleotides changes in the target region of the *vrn-A1* promoter.

Top panel. PCR analysis of transgene segregation (GFP and sgRNA scaffold); lane P, the corresponding plasmid DNA of pGCB or pgRNA-VRNA1#31; lane CS, untransformed ‘Chinese Spring’ wheat plant; numbers 1-23 represent individual T1 plants generated after selfing of the 31-7 primary plant, with the indication of inheritance type of the mutation (biallelic and homozygous) in the bottom of panel.

Bottom panel. Sanger sequencing chromatograms the *vrn-A1* promoter fragments demonstrating the one biallelic and two homozygous combinations of CRISPR/Cas9-induced indels in various T1 plants; PAM sequences are marked in green.

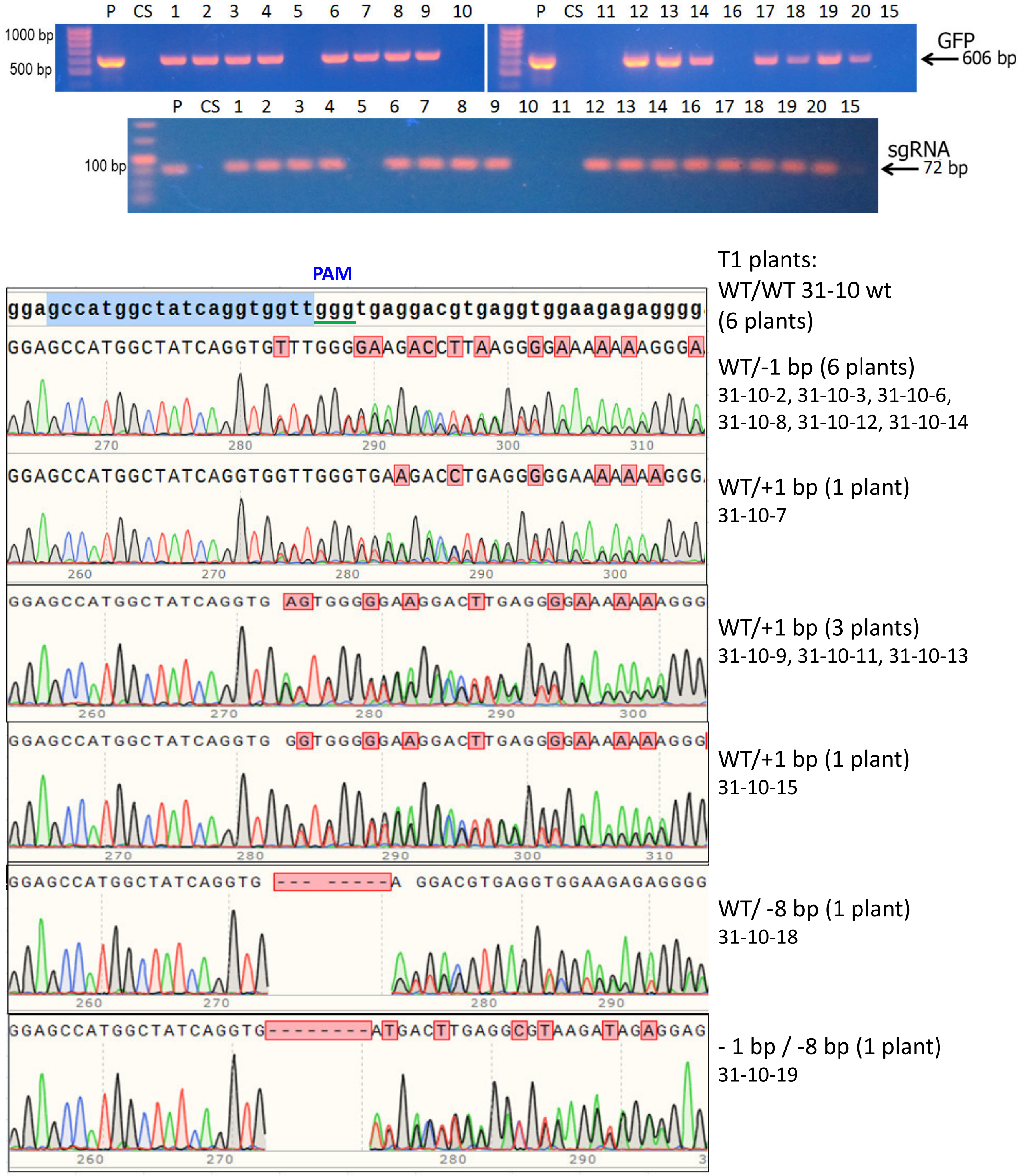

Supplementary Figure S10. Inheritance analysis of the 31-10 primary event carrying the monoallelic nucleotide insertion in the target region of *vrn-A1* promoter.

Top panel. PCR analysis of transgene segregation (*GFP* and sgRNA scaffold); lane P, the corresponding plasmid DNA of pGCB or pgRNA-VRNA1#31; lane CS, untransformed 'Chinese Spring' wheat plant; numbers 1-20 represent individual T1 plants generated after selfing of the 31-10 primary plant.

Bottom panel. Sequencing chromatograms of *vrn-A1* promoter fragment demonstrating new combinations of CRISPR/Cas9-induced indels in various T1 plants; PAM sequences are marked in green.

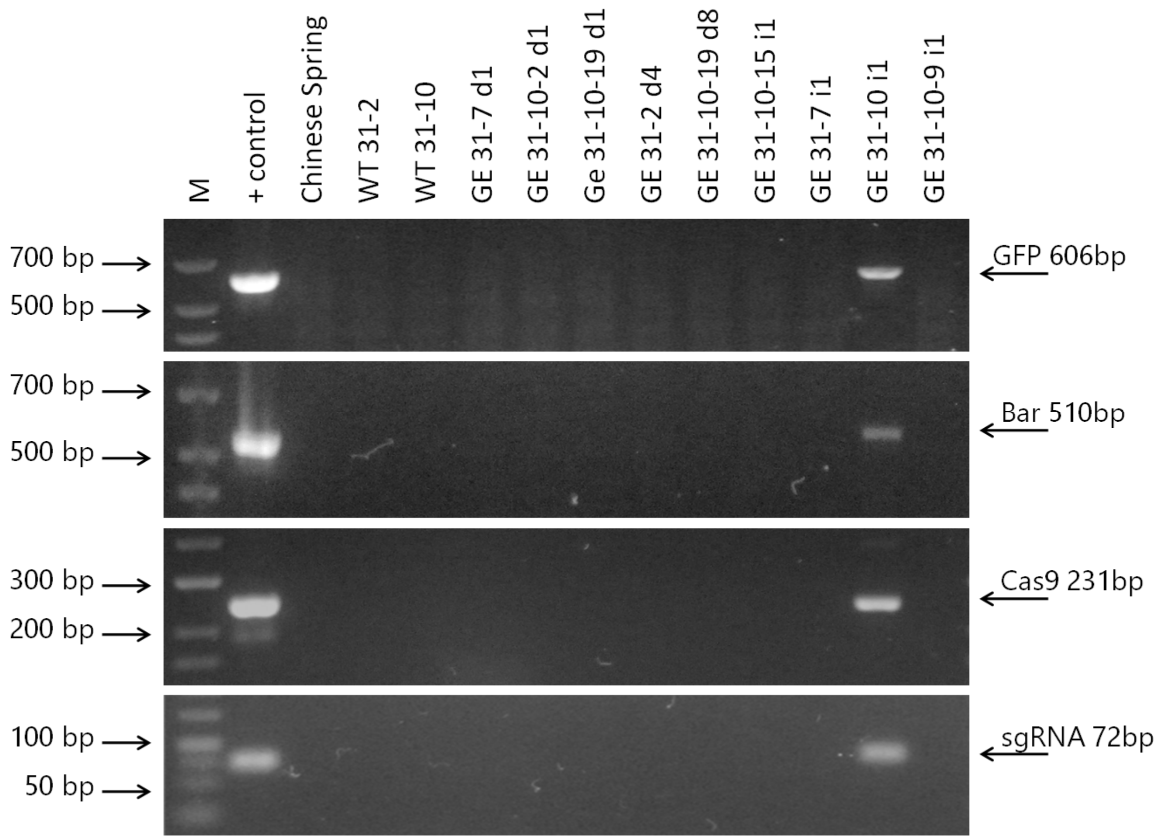

Supplementary Figure S11. PCR analysis for the presence of various transgenic sequences in genome-edited mutated lines used for heading time analysis; lane + control, the corresponding plasmid DNA of pGCB or pgRNA-VRNA1#31 vectors used to produce primary mutated plants; lane Chinese Spring, the DNA of non-transformed wheat cultivar used for genetic modification; the rest of lines, M3 wheat populations carrying various types of CRISPR/Cas9-induced mutations in the target region of *vrn-A1* promoter (for details see Supplementary Table S6). Analyzed M3 populations are transgene-free, except the line GE31-10 i1.

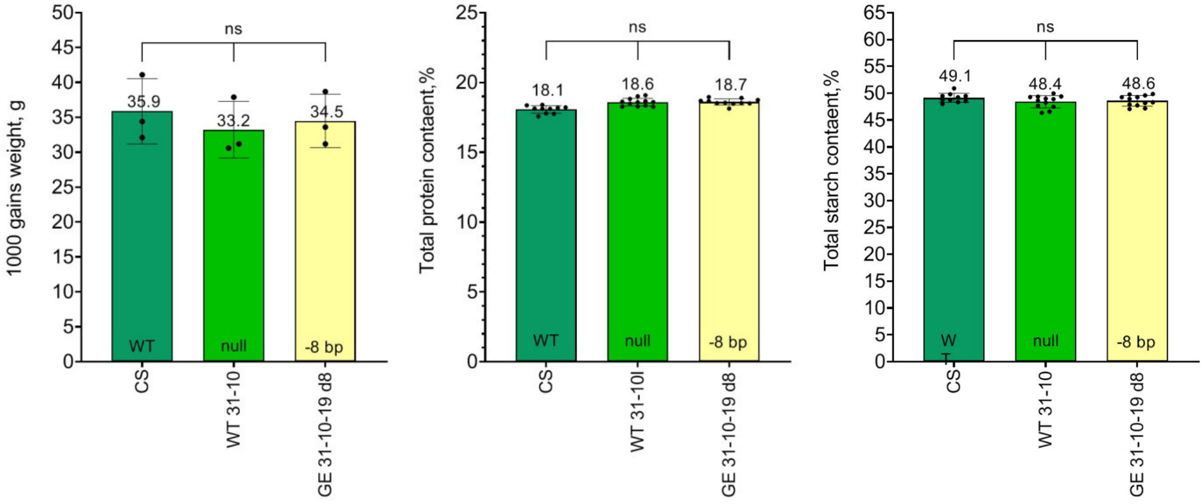

Supplementary Figure S12. Grain quality tests of wheat plants carrying the 8 bp deletion in the promoter region of *vrn-A1* gene (GE 31-10-19 d8), non-transformed 'Chinese Spring' plants (CS) and plants of the transgene-free null-segregant WT 31-10, ns – non significant, Tukey's multiple comparisons test.

Supplementary Table S1. List of primers used in this study

Primer name	Sequence (5' – 3')	Purpose
VRN-A1	CTGAATTCTGAAAGGAAAAATTCTGCTCG<br>ACTGGTACCGAAGGCGTATTGGGGAAC	promoter cloning and sequencing
VRN-A1	AAAAGCACCGACTCGGTGCCACTTTTTCAAGTTGATAACGGA<br>CTAGCCTTATTTTAACTTGCTATTTCTAGCTCTAAAAC	cleavage assay
Cas9	ACGCGAACCTGGATAAGGTC<br>CGATCCTCGTCTCGTACAG	PCR analysis
sgRNA scaffold region	TCGATCCGTAGAAACCAGACG<br>CGACTCGGTGCCACTTTTTC	PCR analysis
GFP	GCGACGTAAACGGCCACAAG<br>CCAGCAGGACCATGTGTGATCG	PCR analysis
Ubi1-BAR	GGATGATGGCATATGCAGCAGCTATATG<br>ACAGCGACCACGCTCTTGAA	PCR analysis
VRN-B1	CTGAATTCATAGTAGTATAAAAAGGACAATTG<br>CTGGTACCACCGAATCAACCAAACAGTG	sequencing
VRN-D1	CTGAATTCGTATAAAAGGAAAATTGTGCTCT<br>ACTGGTACCATCAACCAAACAGCCCCG	sequencing
VRN-A1	TCCACCGAGTCATGTATGGA<br>GAGAACCTTTTCTGCATAAGAA	qPCR
TaWIN	TTTTCTGTGTTCTACTATGAGATCTTGAA<br>AAGTGCATAATTAAACAGAGGTAGTGATG	qPCR

Supplementary Table S2. List of predicted target sequences identified in two regions of the *vrn-A1* promoter (see Figure 1)

sgRNA	Sequence（5′-3′）	orientation
vr-4	AAAAACCCCTCCCCCCCTGCCGG	+
vr-5	CCCCTCCCCCCCTGCCGGAATCC	-
vr-6	CCCTCCCCCCCTGCCGGAATCCT	-
vr-7	CCTCCCCCCCTGCCGGAATCCTC	-
vr-8	CCCCCCCTGCCGGAATCCTCGTT	-
vr-30	ACCCAACCACCTGATAGCCATGG	+
vr-31	CCCAACCACCTGATAGCCATGGC	-
vr-33	CCACCTGATAGCCATGGCTCCGC	-
vr-34	CCTGATAGCCATGGCTCCGCCGC	-
vr-35	CCATGGCTCCGCCGCCTCGCCTC	-
vr-38	CCTCGCCTCCGCCTGCGCCAGTC	-
vr-39	TCGCCTCCGCCTGCGCCAGTCGG	+
vr-40	CCTCCGCCTGCGCCAGTCGGAGT	-
vr-41	CCGCCTGCGCCAGTCGGAGTAGC	-

Supplementary Table S3. Efficiency of genetic co-transformation and targeted mutagenesis in T0 transgenic events of 'Chinese Spring' generated after co-transformation with CRISPR/Cas9 encoding vectors

sgRNA expressing vector *	Number of bombarded explant	Number of GFP positive/ herbicide resistant T0 plants	Number of PCR positive T0 plants		Co-transformation efficiency (Cas9+sgRNA), %	Number of T0 events with mutation	Mutagenesis frequency, %
			Cas9	sgRNA			
pgRNA-VRNA1#30	652	9	9	9	1.4	0	0.0
pgRNA-VRNA1#31	603	10	10	8	1.3	4	0.7
pgRNA-VRNA1#34	500	6	6	6	1.2	0	0.0

* In all experiments the vector encoding specific sgRNA was co-bombarded into morphogenic tissues with the plasmid pGCB carrying sequences for the expression of *Cas9*, *GFP* and *bar* genes

Supplementary Table S4 Summary of inheritance of CRISPR/Cas9-induced indels and segregation of transgenes in T1 plants produced after self pollination of four primary mutated wheat plants*

T0 plant	Number of T1 plants tested	Segregation of transgenes **				Segregation of mutation ***			
		vector	Observed transgene segregation ratio (positive:negative	χ^2 value for the expected segregation ratio (positive:negative)		Observed segregation ratio (null:monoallelic: homozygous)	χ^2 value for the expected segregation ratio 1 [null] : 2 [monoalleic mutation] : 1 [mutation]		
				3:1	15:1		null	monoallelic	homozygous
31-1	10	pGCB	8 : 2	0.13	3.23	1 : 7 : 2	1.20	1.6	0.13
		pgRNA-VRNA1#31	10 : 0	-	-				
31-2	23	pGCB	5 : 18	34.8 ns	203.6 ns	5 : 13 : 5	0.13	0.39	0.13
		pgRNA-VRNA1#31	5 : 18	34.8 ns	203.6 ns				
31-10	20	pGCB	15 :5	0.00	15.0 ns	Not available due to himerism of the primatry T0 plant			
		pgRNA-VRNA1#31	15 :5	0.00	15.0 ns				
						(homozygous for mutation 1 : biallelic : homozygous for mutation 2)	homozygous 1	biallelic	homozygous 2
31-7	13	pGCB	8 : 5	1.26	23.02 ns	4 : 6 : 3	0.23	0.08	0.03
		pgRNA-VRNA1#31	8 : 5	1.26	23.02 ns				

* the presence of transgenic sequences in the genome of individual plants was assessed by PCR and GFP fluorescence in pollen

** the segregation of transgenes was analyzed using χ^2 value for the expected Mendelian 1 [transgene negative] : 3 [transgene positive] ratio; if the χ^2 value is above 3.84 ($P > 0.05$) the observed segregation ratio is not significantly different from the expected ratio

*** indels segregation was confirmed by sequencing of amplicons corresponding to the target region of *vrn-A1* promoter and analyzed using χ^2 value for the expected Mendelian 1 [null] : 2 [monoallelic mutation] : 1 [homozygous mutation] ratio or 1 [homozygous mutation 1] : 2 [bialleic mutations] : 1 [homozygous mutation 2] ratio; if the χ^2 value is above 3.84 ($P > 0.05$) the observed segregation ratio is not significantly different from the expected ratio

Supplementary Table S5. Summary of inheritance of CRISPR/Cas9-induced indels and segregation of transgenes in T2 plants produced after self pollination of #31-10 T1 plants *

T1 plant	Transgene status*	Type of indels in the T1 plant	T2 plants analyzed	T2 plants with the various type of indel segregation**						T2 plants not inherited transgenes (GM-free)***		
				WT	χ^2 1:3	hetero	χ^2 2:2	homo	χ^2 1:3	All	χ^2 1:3	with homozygous indel segregation
31-10-2	Cas9+ sgRNA	AGGAGTGGGTT-GTGGACTATCGGTACCGAGGCG<br>AGGAGTGGGTTGGTGGACTATCGGTACCGAGGCG	18	4	0.07	9	0.00	5	0.07	5	0.07	2
31-10-7	Cas9+ sgRNA	AGGAGTGGGTTGGTGGACTATCGGTACCGAGGCG<br>AGGAGTGGGTTGTGTGGACTATCGGTACCGAGGCG	14	5	0.86	5	1.14	4	0.10	3	0.09	0
31-10-9	Cas9+ sgRNA	AGGAGTGGGTTGGTGGACTATCGGTACCGAGGCG<br>AGGAGTGGGTTGAGTGGACTATCGGTACCGAGGCG	10	1	1.20	6	0.40	3	0.13	3	0.13	1
31-10-11	free		10	3	0.13	6	0.40	1	1.20	10	-	1
31-10-15	free	AGGAGTGGGTTGGTGGACTATCGGTACCGAGGCG<br>AGGAGTGGGTTGGGTGGACTATCGGTACCGAGGCG	19	3	0.86	9	0.05	7	1.42	19	-	7
31-10-19	Cas9+ sgRNA	AGGAGTGGGTT-GTGGACTATCGGTACCGAGGCG<br>AGGA--------GTGGACTATCGGTACCGAGGCG	19	-		10	0.05	9<br>7 plants with -1 bp<br>2 plants with -8 bp	1.42<br><br>2.12	7	1.42	3<br>2 plants with -1 bp<br>1 plant with -8 bp

* presence of transgenic sequences in the genome of individual plants was assessed by PCR and GFP fluorescence in pollen

** indel segregation was confirmed by sequencing of amplicons corresponding to the target region of *vrn-A1* promoter, the indel segregation was analysed using χ2 value for the expected Mendelian 1 [null] : 2 [monoalleic mutation] : 1 [homozygous mutation] ratio, if the χ^2 value is above 3.84 ($P > 0.05$) the observed segregation ratio is not significantly different from the expected ratio

*** the segregation of transgenes was analyzed using χ^2 value for the expected Mendelian 1 [transgene negative] : 3 [transgene positive] ratio, if the χ^2 value is above 3.84 ($P > 0.05$) the observed segregation ratio is not significantly different from the expected ratio

Supplementary Table S6. List of the T3 (M3) population used for greenhouse-based analysis of the heading time

Mutation type		Line	Plants analyzed*	Transgene status**
WT	AGGAGTGGGTTGGTGGACTATCGGTACCGAGGCG	Chinese Spring	61	FREE
null	AGGAGTGGGTTGGTGGACTATCGGTACCGAGGCG	WT 31-2<br>WT 31-10	27<br>45	FREE
-1 bp	AGGAGTGGGTT-GTGGACTATCGGTACCGAGGCG	GE 31-7 d1<br>GE 31-10-2 d1<br>Ge 31-10-19 d1	51<br>24<br>48	FREE
-4 bp	AGGAGTG----GGTGGACTATCGGTACCGAGGCG	GE 31-2 d4	59	FREE
-8 bp	AGGA--------GTGGACTATCGGTACCGAGGCG	GE 31-10-19 d8	71	FREE
+1 bp	AGGAGTGGGTTGGGTGGACTATCGGTACCGAGGCG	GE 31-10-15 i1	45	FREE
+1 bp	AGGAGTGGGTTGCGTGGACTATCGGTACCGAGGCG	GE 31-7 i1	48	FREE
+1 bp	AGGAGTGGGTTGTGTGGACTATCGGTACCGAGGCG	GE 31-10 i1	45	Cas9+sgRNA
+1 bp	AGGAGTGGGTTGAGTGGACTATCGGTACCGAGGCG	GE 31-10-9 i1	24	FREE

* only plants carrying homozygous mutated alleles were analyzed

** absence of transgenic sequences in the genome was assessed by PCR and GFP fluorescence in pollen

Supplementary Table S7. Tukey's multiple comparisons test for the experimental lines subjected to the heading time analysis (see Supplementary Table S6)

	Mean 1	Mean 2	N1	N2	Mean Diff,	95,00% CI of diff,	Significant?	Summary	Adjusted P Value
SC vs. WT 31-2	63,75	65,30	61	27	-1,542	-3,406 to 0,3219	No	ns	0,2214
SC vs. GE 31-2 d4	63,75	64,61	61	59	-0,8561	-2,329 to 0,6165	No	ns	0,7538
SC vs. WT 31-10	63,75	63,56	61	45	0,1985	-1,386 to 1,783	No	ns	>0,9999
SC vs. GE 31-10-19 d8	63,75	61,35	61	72	2,407	1,003 to 3,810	Yes	****	<0,0001
SC vs. GE 31-7 d1	63,75	64,73	61	51	-0,9714	-2,502 to 0,5588	No	ns	0,6347
SC vs. GE 31-10-19 d1	63,75	64,31	61	48	-0,5584	-2,114 to 0,9976	No	ns	0,9904
SC vs. GE 31-10-2 d1	63,75	64,17	61	24	-0,4126	-2,356 to 1,531	No	ns	>0,9999
SC vs. GE 31-7 i1	63,75	64,96	61	45	-1,201	-2,786 to 0,3833	No	ns	0,3494
SC vs. GE 31-10 i1	63,75	63,87	61	45	-0,1126	-1,697 to 1,472	No	ns	>0,9999
SC vs. GE 31-10-9 i1	63,75	63,30	61	30	0,4541	-1,344 to 2,252	No	ns	0,9996
SC vs. GE 31-10-15 i1	63,75	64,04	61	45	-0,2903	-1,875 to 1,294	No	ns	>0,9999
WT 31-2 vs. GE 31-2 d4	65,30	64,61	27	59	0,6861	-1,188 to 2,560	No	ns	0,9887
WT 31-2 vs. WT 31-10	65,30	63,56	27	45	1,741	-0,2225 to 3,704	No	ns	0,1401
WT 31-2 vs. GE 31-10-19 d8	65,30	61,35	27	72	3,949	2,129 to 5,769	Yes	****	<0,0001
WT 31-2 vs. GE 31-7 d1	65,30	64,73	27	51	0,5708	-1,349 to 2,490	No	ns	0,9981
WT 31-2 vs. GE 31-10-19 d1	65,30	64,31	27	48	0,9838	-0,9563 to 2,924	No	ns	0,8834
WT 31-2 vs. GE 31-10-2 d1	65,30	64,17	27	24	1,130	-1,133 to 3,392	No	ns	0,8939
WT 31-2 vs. GE 31-7 i1	65,30	64,96	27	45	0,3407	-1,622 to 2,304	No	ns	>0,9999
WT 31-2 vs. GE 31-10 i1	65,30	63,87	27	45	1,430	-0,5336 to 3,393	No	ns	0,4144
WT 31-2 vs. GE 31-10-9 i1	65,30	63,30	27	30	1,996	-0,1430 to 4,136	No	ns	0,0940
WT 31-2 vs. GE 31-10-15 i1	65,30	64,04	27	45	1,252	-0,7113 to 3,215	No	ns	0,6280
GE 31-2 d4 vs. WT 31-10	64,61	63,56	59	45	1,055	-0,5415 to 2,651	No	ns	0,5731
GE 31-2 d4 vs. GE 31-10-19 d8	64,61	61,35	59	72	3,263	1,847 to 4,679	Yes	****	<0,0001
GE 31-2 d4 vs. GE 31-7 d1	64,61	64,73	59	51	-0,1153	-1,657 to 1,427	No	ns	>0,9999
GE 31-2 d4 vs. GE 31-10-19 d1	64,61	64,31	59	48	0,2977	-1,270 to 1,865	No	ns	>0,9999
GE 31-2 d4 vs. GE 31-10-2 d1	64,61	64,17	59	24	0,4435	-1,509 to 2,396	No	ns	0,9999
GE 31-2 d4 vs. GE 31-7 i1	64,61	64,96	59	45	-0,3454	-1,942 to 1,251	No	ns	>0,9999
GE 31-2 d4 vs. GE 31-10 i1	64,61	63,87	59	45	0,7435	-0,8526 to 2,340	No	ns	0,9321
GE 31-2 d4 vs. GE 31-10-9 i1	64,61	63,30	59	30	1,310	-0,4982 to 3,119	No	ns	0,4228
GE 31-2 d4 vs. GE 31-10-15 i1	64,61	64,04	59	45	0,5657	-1,030 to 2,162	No	ns	0,9914
WT 31-10 vs. GE 31-10-19 d8	63,56	61,35	45	72	2,208	0,6758 to 3,741	Yes	***	0,0002
WT 31-10 vs. GE 31-7 d1	63,56	64,73	45	51	-1,170	-2,819 to 0,4795	No	ns	0,4577
WT 31-10 vs. GE 31-10-19 d1	63,56	64,31	45	48	-0,7569	-2,430 to 0,9165	No	ns	0,9443
WT 31-10 vs. GE 31-10-2 d1	63,56	64,17	45	24	-0,6111	-2,650 to 1,427	No	ns	0,9980
WT 31-10 vs. GE 31-7 i1	63,56	64,96	45	45	-1,400	-3,100 to 0,3002	No	ns	0,2276
WT 31-10 vs. GE 31-10-7 i1	63,56	63,87	45	45	-0,3111	-2,011 to 1,389	No	ns	>0,9999
WT 31-10 vs. GE 31-10-9 i1	63,56	63,30	45	30	0,2556	-1,645 to 2,156	No	ns	>0,9999
WT 31-10 vs. GE 31-10-15 i1	63,56	64,04	45	45	-0,4889	-2,189 to 1,211	No	ns	0,9986
GE 31-10-19 d8 vs. GE 31-7 d1	61,35	64,73	72	51	-3,378	-4,854 to -1,902	Yes	****	<0,0001
GE 31-10-19 d8 vs. GE 31-10-19 d1	61,35	64,31	72	48	-2,965	-4,468 to -1,463	Yes	****	<0,0001
GE 31-10-19 d8 vs. GE 31-10-2 d1	61,35	64,17	72	24	-2,819	-4,720 to -0,9186	Yes	****	<0,0001
GE 31-10-19 d8 vs. GE 31-7 i1	61,35	64,96	72	45	-3,608	-5,141 to -2,076	Yes	****	<0,0001
GE 31-10-19 d8 vs. GE 31-10 i1	61,35	63,87	72	45	-2,519	-4,052 to -0,9869	Yes	****	<0,0001
GE 31-10-19 d8 vs. GE 31-10-9 i1	61,35	63,30	72	30	-1,953	-3,705 to -0,2003	Yes	*	0,0146
GE 31-10-19 d8 vs. GE 31-10-15 i1	61,35	64,04	72	45	-2,697	-4,230 to -1,165	Yes	****	<0,0001
GE 31-7 d1 vs. GE 31-10-19 d1	64,73	64,31	51	48	0,4130	-1,209 to 2,035	No	ns	0,9996
GE 31-7 d1 vs. GE 31-10-2 d1	64,73	64,17	51	24	0,5588	-1,437 to 2,555	No	ns	0,9989
GE 31-7 d1 vs. GE 31-7 i1	64,73	64,96	51	45	-0,2301	-1,879 to 1,419	No	ns	>0,9999
GE 31-7 d1 vs. GE 31-10 i1	64,73	63,87	51	45	0,8588	-0,7906 to 2,508	No	ns	0,8636
GE 31-7 d1 vs. GE 31-10-9 i1	64,73	63,30	51	30	1,425	-0,4301 to 3,281	No	ns	0,3286
GE 31-7 d1 vs. GE 31-10-15 i1	64,73	64,04	51	45	0,6810	-0,9684 to 2,330	No	ns	0,9711

	Mean 1	Mean 2	N1	N2	Mean Diff,	95,00% CI of diff,	Significant?	Summary	Adjusted P Value
GE 31-10-19 d1 vs. GE 31-10-2 d1	64,31	64,17	48	24	0,1458	-1,870 to 2,162	No	ns	>0,9999
GE 31-10-19 d1 vs. GE 31-7 i1	64,31	64,96	48	45	-0,6431	-2,316 to 1,030	No	ns	0,9834
GE 31-10-19 d1 vs. GE 31-10 i1	64,31	63,87	48	45	0,4458	-1,228 to 2,119	No	ns	0,9993
GE 31-10-19 d1 vs. GE 31-10-9 i1	64,31	63,30	48	30	1,013	-0,8644 to 2,889	No	ns	0,8333
GE 31-10-19 d1 vs. GE 31-10-15 i1	64,31	64,04	48	45	0,2681	-1,405 to 1,941	No	ns	>0,9999
GE 31-10-2 d1 vs. GE 31-7 i1	64,17	64,96	24	45	-0,7889	-2,827 to 1,250	No	ns	0,9824
GE 31-10-2 d1 vs. GE 31-10 i1	64,17	63,87	24	45	0,3000	-1,738 to 2,338	No	ns	>0,9999
GE 31-10-2 d1 vs. GE 31-10-9 i1	64,17	63,30	24	30	0,8667	-1,342 to 3,075	No	ns	0,9804
GE 31-10-2 d1 vs. GE 31-10-15 i1	64,17	64,04	24	45	0,1222	-1,916 to 2,161	No	ns	>0,9999
GE 31-7 i1 vs. GE 31-10 i1	64,96	63,87	45	45	1,089	-0,6113 to 2,789	No	ns	0,6214
GE 31-7 i1 vs. GE 31-10-9 i1	64,96	63,30	45	30	1,656	-0,2453 to 3,556	No	ns	0,1590
GE 31-7 i1 vs. GE 31-10-15 i1	64,96	64,04	45	45	0,9111	-0,7891 to 2,611	No	ns	0,8393
GE 31-10 i1 vs. GE 31-10-9 i1	63,87	63,30	45	30	0,5667	-1,334 to 2,468	No	ns	0,9981
GE 31-10 i1 vs. GE 31-10-15 i1	63,87	64,04	45	45	-0,1778	-1,878 to 1,522	No	ns	>0,9999
GE 31-10-9 i1vs. GE 31-10-15 i1	63,30	64,04	30	45	-0,7444	-2,645 to 1,156	No	ns	0,9807
